# A Review on Engineering Transition Metal Compound Catalysts to Accelerate the Redox Kinetics of Sulfur Cathodes for Lithium–Sulfur Batteries

**DOI:** 10.1007/s40820-023-01299-9

**Published:** 2024-01-29

**Authors:** Liping Chen, Guiqiang Cao, Yong Li, Guannan Zu, Ruixian Duan, Yang Bai, Kaiyu Xue, Yonghong Fu, Yunhua Xu, Juan Wang, Xifei Li

**Affiliations:** 1 Shaanxi Key Laboratory of Nanomaterials and Nanotechnology, Xi’an University of Architecture and Technology, Xi’an, 710055 People’s Republic of China; 2 Institute of Advanced Electrochemical Energy and School of Materials Science and Engineering, Xi’an University of Technology, Xi’an, 710048 People’s Republic of China; 3 Yulin University, Yulin, 719000 People’s Republic of China; 4 School of Materials Science and Engineering, Fuzhou University, Fuzhou, 350108 People’s Republic of China

**Keywords:** Lithium–sulfur battery, Redox kinetic, Transition metal compounds catalyst, Multiple metals/anions

## Abstract

Engineering transition metal compounds (TMCs) catalysts with excellent adsorption-catalytic ability has been one of the most effective strategies to accelerate the redox kinetics of sulfur cathodes. Herein, this review focuses on engineering TMCs catalysts by cation doping/anion doping/dual doping, bimetallic/bi-anionic TMCs, and TMCs-based heterostructure composites. It is obvious that introducing cations/anions to TMCs or constructing heterostructure can boost adsorption-catalytic capacity by regulating the electronic structure including energy band, *d*/*p*-band center, electron filling, and valence state. Moreover, the electronic structure of doped/dual-ionic TMCs are adjusted by inducing ions with different electronegativity, electron filling, and ion radius, resulting in electron redistribution, bonds reconstruction, induced vacancies due to the electronic interaction and changed crystal structure such as lattice spacing and lattice distortion. Different from the aforementioned two strategies, heterostructures are constructed by two types of TMCs with different Fermi energy levels, which causes built-in electric field and electrons transfer through the interface, and induces electron redistribution and arranged local atoms to regulate the electronic structure. Additionally, the lacking studies of the three strategies to comprehensively regulate electronic structure for improving catalytic performance are pointed out. It is believed that this review can guide the design of advanced TMCs catalysts for boosting redox of lithium sulfur batteries.

## Highlights

- The representatively engineering strategies of cations/anions doping, bimetallic/bi-anionic transition metal compounds and heterostructure composites catalysts for lithium sulfur batteries are comprehensively reviewed.
- The promoted mechanism of catalytic performance by regulating electronic structure is focused on, including energy band, electron filling,* d*/*p*-band center, valence state.
- The superiority of the modified transition metal compounds is comprehensively summarized.

## Graphical abstract

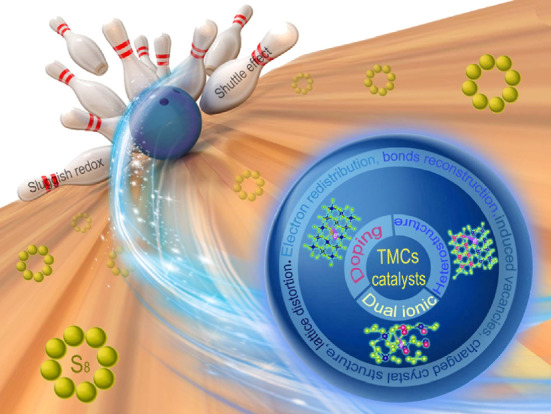

## Introduction

Lithium-ion batteries are the most successful energy storage system developed in the past 30 years for their relatively high energy density and cycle stability. However, the limited theoretical energy density (420 Wh kg^−1^) of lithium ion batteries does not meet the requirements of some applications such as electric vehicles, and it has been urgent to develop high-performance batteries with higher energy density [1]. Lithium–sulfur batteries (LSBs) have been widely concerned since 2009, when Nazar adopted mesoporous carbon as sulfur host, bringing its development possibility for high theoretical specific capacity of 1675 mAh g^−1^ and energy density of 2600 Wh kg^−1^ [2, 3]. Liquid electrolyte solves the sluggish kinetic of solid–solid reaction of LSBs from the initial S_8_ directly to the final reaction product of the Li_2_S. However, challenges of LSBs come along with liquid electrolytes. Various intermediates lithium polysulfides (LiPSs, Li_2_S_*n*_, 2 ≤ *n* ≤ 8) are formed during multi-step charge–discharge process, and their solubility in the electrolyte causes their department from cathode materials [4]. The infamous shuttle effect of LiPSs is formed in the electric field, which leads to the loss of sulfur species and capacity fading [5].

Since 2009, carbon materials represented by porous carbon have been used to physically adsorb LiPSs to mitigate the shuttle effect [6, 7]. Then, polar materials including doped carbon materials and various transition metal compounds (TMCs) with chemical interaction with LiPSs were introduced to sulfur cathodes, interlayers and modified separators [8, 9]. However, physical confinement with weak interaction and chemisorption strategy with limited adsorption sites are still not ideal to solve shuttle effect [2, 10]. Additionally, the chemisorption for LiPSs is not a determinant of the cathode performance [11]. The conversion of S_8_ → Li_2_S_8_ is relatively easy and spontaneous, while the long-chain LiPSs are easily dissolved, and the transformation of Li_2_S_4_ to Li_2_S_2_/Li_2_S requires high activation energy, which leads to the accumulation of LiPSs, and aggravate the shuttling effect. In addition, the transition from Li_2_S_2_ to Li_2_S is slow in charge transfer kinetics due to its insulating properties, which is the most difficult stage and regarded as the rate-limiting step during discharging [12, 13]. During the charging stage, the oxidation of Li_2_S needs to overcome high energy barrier due to its slow kinetics, leading to the high overpotential [14]. Therefore, the key factor of the shuttle effect is the sluggish reaction kinetics of LiPSs/Li_2_S in addition to the solubility of LiPSs [10]. If LiPSs is not quickly converted, the active sites will be occupied, resulting in the reduction of adsorption effect [12]. Therefore, catalytic effect is regarded as the more promising strategy to fundamentally solve the problem [10]. The catalysts can not only anchor LiPSs, but also boost the conversion from LiPSs to Li_2_S and the oxidation of Li_2_S, shorten the existence time of LiPSs and reduce the accumulation of LiPSs, effectively alleviating the shuttle effect [5].

## Advanced TMCs Catalysts

### Characteristics of Catalysts for Sulfur Cathodes

Efficient catalysts for sulfur cathodes should possess the following advantages: high conductivity, rapid electron/ion transfer, moderate adsorption capacity, excellent catalytic activity and abundant active sites to promote the redox of LiPSs/Li_2_S [15]. The catalytic capacity of sulfur catalysts is firstly related to their conductivity. The LSBs involve multi-electron reaction, and the high conductivity will promote the electrochemical reaction [16]. Secondly, catalysis requires the adsorption of LiPSs to active sites. If LiPSs are weakly adsorbed or cannot fully contact with catalytic sites, the catalytic effect cannot be fully played [12]. The interaction of polar materials and LiPSs also promotes charge transfer between them, provides Li_2_S nucleation sites and regulates its uniform deposition as well as Li_2_S decomposition [16, 17]. In the meanwhile, the transferred electrons determine the strength of the interaction [18]. On the other hand, too strong adsorption for LiPSs will occupy active sites and hinder further conversion of LiPSs [12, 19]. Most importantly, the intrinsic catalytic activity is the fundamental factor for the catalytic effect [13]. Additionally, the fast diffusion rate of Li^+^ not only promotes the electrochemical reaction, but also reflects fast electrochemical reaction kinetics [20, 21]. Some catalysts can even adjust the electronic structure of adsorbed Li_2_S, conferring an insulator-to-metal transition to improve the conductivity of Li_2_S [22].

### Advantages of TMCs Catalysts for LSBs

At present, tuning reaction kinetics of LSBs has been extensively investigated through electrolyte mediators, non-metal catalyst, and nanostructured metal-based catalysts. Among them, electrolyte mediators can effectively manipulate the conversion behavior of sulfur and Li_2_S such as reaction pathway, types of LiPSs, voltage polarization, Li_2_S deposition morphologies, and Li_2_S activation by controlling solvent species, LiPS dissolvability, salt species and concentration, addition of electrolyte additives as well as solid-state electrolytes. However, the electrolytic liquid system is complicated, and there are contradictions with the compatibility of lithium anodes, ionic conductivity, and viscosity [23]. Non-metal materials like black phosphorus and functionalized carbon possess significant advantages in high specific surface area and light weight, the former is conducive to increasing the active area to promote the electrochemical reaction processes, and the latter is beneficial to improving the mass energy density of the battery. Unfortunately, black phosphorus only presents strong adsorption ability for LiPSs at the edge, but weak in the plane, showing the characteristics of edge selective catalysis [24]. Similarly, the polar sites on the surface of modified carbon materials are limited. Moreover, the non-metal-Li bond formed between doped anions of functionalized carbon and Li of LiPSs does not play a key role for LiPSs adsorption as S-binding between metal atoms in TMCs and S atoms in LiPSs [25]. Additionally, metal-based catalysts include supported single atom catalysts (SACs), metallic nanostructures, and TMCs. SACs with theoretical 100% atomic utilization, unsaturated coordination environment, and unique electronic structure are expected to achieve efficient catalysis for LSBs [1]. However, SACs still suffer from the problem of poor stability, easy aggregation and low load due to their high surface energy. Nanostructured metal materials with sulfiphilicity, excellent conductivity and catalytic activity are another kind of excellent catalysts for LSBs [26]. Alloys can effectively regulate its electronic structure with different metal elements, such as *d*-band center, thus improving the catalytic activity [27, 28]. However, the enhanced catalytic properties which are closely related to optimized electronic structure via compositional design are rarely in depth studied in LSBs. Simultaneously, the nano-alloy catalysts are lack of the chemical interaction with LiPSs through Li-non-metal bonds, and could not adjusted by non-metal ions. In contrast, TMCs have been extensively studied in LSBs, including metal oxides, sulfides, nitrides, carbides, phosphide, selenides, metal–organic framework (MOFs) due to excellent chemisorption and catalytic effect. Qian revealed that Co-based compounds followed the order of CoP > Co_4_N > CoS_2_ > Co_3_O_4_ to accelerate the redox kinetics of LSBs. The essential reason is that the *p*-band center of CoP was upshifted obviously, reducing the energy gap between the *d*-band center of Co and the *p*-band center [29]. Metal selenides exhibit similar crystal structure and polarity characteristics to sulfides, while much higher conductivity and catalytic activity [30]. All the metal-based catalysts contain *d* orbitals of transition metals that can be hybridized with the *p* orbital of S of LiPSs/Li_2_S, thereby reducing the reaction barrier by changing the electronic structure of LiPSs/Li_2_S [31, 32]. Moreover, the *d* and *p* orbitals of TMCs are hybridized with the *p* and *s* orbitals of S and Li in LiPSs, forming metal-S bonds and Li-nonmetallic bonds, which makes TMCs possess larger modulation space of electronic structure to anchor and catalyze LiPSs more effectively through introducing metal ions or anions [33, 34].

Most importantly, it is difficult for single-component catalysts with single electron donor or acceptor nature to catalyze multi-step conversions of sulfur cathodes, and different metal cations of TMCs can provide different binding strength, binding preferences and catalytic functions for diverse LiPSs. It is of great significance to construct TMCs catalyst with more components for addressing the complex redox of LSBs through enriching active sites, designing multi-function and synergy effect [23, 35, 36]. Additionally, TMCs catalysts with multiple metals/anions can optimize intrinsic catalytic activity with tuned electronic structure, and show huge room for regulation. Consequently, more and more TMCs catalysts with multiple metal ions or anions are proposed to enrich active sites, improve conductivity, chemisorption and catalytic activity. The design principles, structures and properties of various bimetallic compounds used as sulfur host materials, doping modification for carbon materials, C_3_N_4_ and MXenes, and advances in heterostructure optimization for sulfur cathodes, interlayers and lithium anodes have been reviewed [37–39]. However, the ways of introducing more cations/anions to modify TMCs, and the promoted mechanism have not been reviewed. Therefore, engineering strategies of TMCs to boost their catalytic effect as the focus are reviewed, and the unsolved problems as well as the further research is also prospected.

## Engineering TMCs Catalysts

**Fig. 1:**
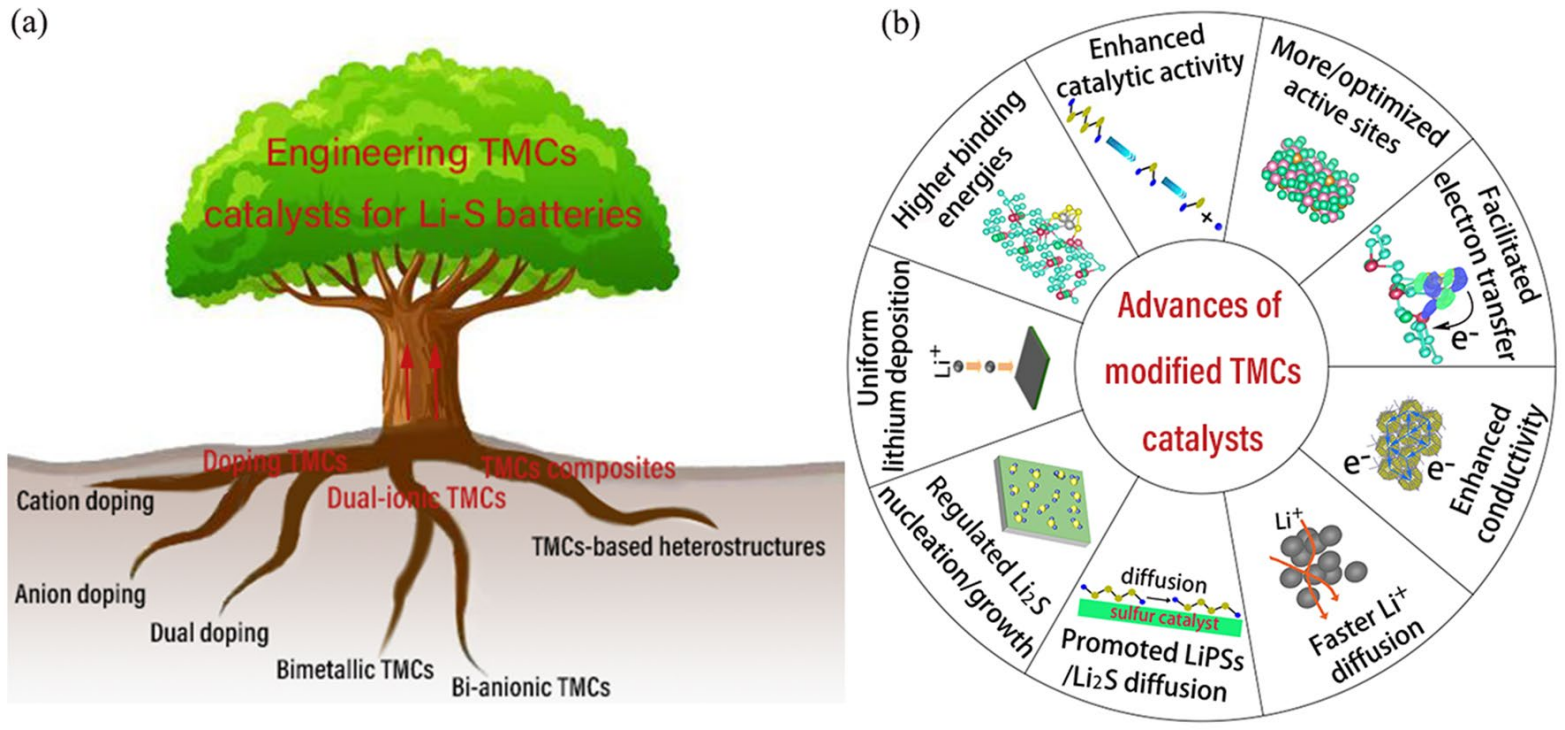
**a** Strategies, **b** advances of modified transition metal compounds (TMCs) catalysts with multi-cations/anions in LSBs

TMCs nanomaterials involve electronic interactions between metal ions and anions, and the electron density will be redistributed after the metal is coordinated with the anion, thus giving TMCs materials adjustable catalytic activity. Regulating the *d*-*p* orbital hybridization state of TMCs catalysts and LiPSs/Li_2_S by changing the electron structure of TMCs, is the essence to improve the catalytic activity. Engineering TMCs catalysts with multiple cations/anions can optimize intrinsic catalytic activity with tuned electronic structure through doping modification, constructing dual-ionic TMCs and TMCs-based heterostructure composites (Fig. 1a). These modification will enable TMCs catalysts with the superiority of enhanced catalytic activity, higher binding energies, more active sites, etc. (Fig. 1b).

### Doping Modification

Heteroatom doping can not only enhance the electrical conductivity of TMCs, afford more chemical anchoring sites and higher adsorption energies for LiPSs, but also improve the charge transfer compared with undoped ones [40, 41]. The catalytic activity is thus enhanced, improving the sulfur utilization and electrochemical performance. According to the types of doped ions, doping modification includes cation doping, anion doping and dual doping.

#### Cation Single-Doping Modification

**Fig. 2:**
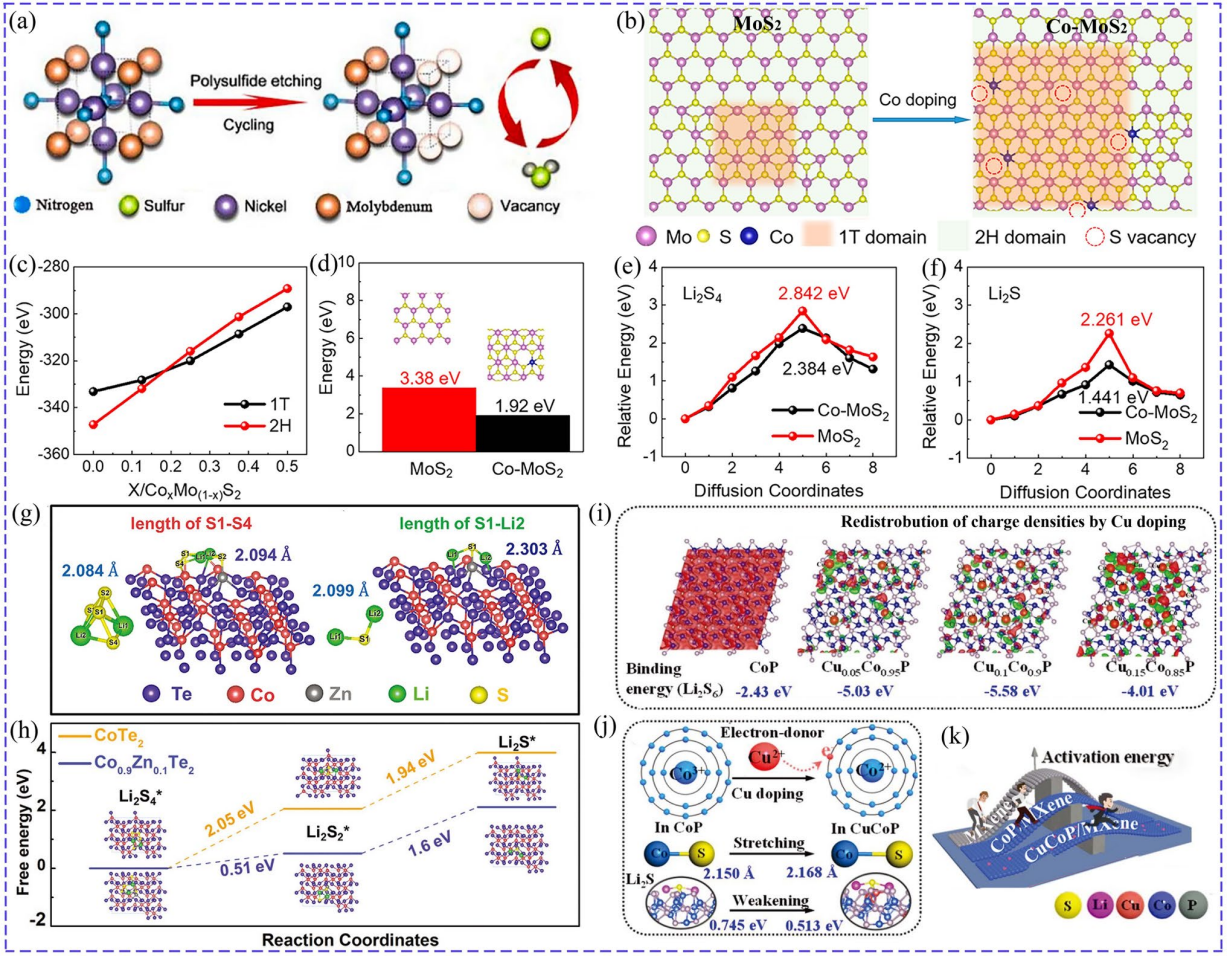
**a** Schematic of in situ etching Mo-doped Ni_3_N by LiPSs [44]. Copyright: 2022, Elsevier. **b** Schematic of evolution of Co-doped MoS_2_. **c** Relationship of the formation energies of 1 T, 2H MoS_2_ and doped Co content. **d** Influence of Co doping on the formation energies of sulfur vacancies in MoS_2_. The catalytic effect of Co-doped MoS_2_: **e** Li_2_S_4_ decomposition and **f** Li_2_S decomposition [45]. Copyright: 2021, American Chemical Society. Effect of Zn doping on the catalytic effect of CoTe_2_: **g** S–S bond length of Li_2_S_4_ and S–Li bond length of Li_2_S. **h** Gibbs free energies [47]. Copyright: 2022, John Wiley and Sons. Effect of Cu-doped CoP: **i** unbalanced charge densities induced by Cu doping. **j** Schematic of electron transfer and variation of bond length, **k** Comparison of LiPSs/Li_2_S conversion barrier [54]. Copyright: 2019, John Wiley and Sons

Since cations usually act as active sites, adjusting their electron structure is essential to improve catalytic activity [42]. For example, surface defect was introduced into MoS_2_ to enrich active sites by Ni doping, which promoted Li^+^/e^−^ transfer, chemisorption and catalytic effect of Ni-doped MoS_2_ toward LiPSs, improving the redox kinetics of LSBs. This rendered the sulfur cathodes with Ni-MoS_2_ achieve a higher specific capacity of 1343.6 mAh g^−1^ with retained capacity of 800 mAh g^−1^ at 0.2C for 100 cycles than that of undoped MoS_2_ (1287.8 mAh g^−1^, 678.3 mAh g^−1^) [43]. Min found that the lattice spacing of Ni_0.2_Mo_0.8_N was larger than that of Ni_3_N and Mo_2_N due to the changed *d*-band position, improving the delocalization of electrons and lithium ions transfer. Additionally, because the Mo was more positive at the corners than Ni (Fig. 2a), Mo was partially etched by LiPSs during cycling, which generated much vacancies around Ni, accelerating charge transfer and LiPSs conversion. Furthermore, a built-in electric field was formed between the Ni_0.2_Mo_0.8_N modified separator and lithium metal due to the higher surface potential of Ni_0.2_Mo_0.8_N, which was beneficial for preventing the LiPSs diffusion and promoting Li^+^ transmission [44]. Co was also doped into MoS_2_, rendering the 2H MoS_2_ transform into 1 T phase with sulfur vacancies, as illustrated in Fig. 2b. And it was easy for Co-doped MoS_2_ to stabilize the 1 T phase and sulfur vacancies due to their lower formation energy of sulfur vacancies with 1.92 eV than MoS_2_ (3.38 eV) (Fig. 2c, d). The electron-rich Co provided electrons for S and promoted the electron transfer, thus effectively boosting the adsorption capacity and catalytic activity, and reducing the decomposition energy barriers of Li_2_S_4_/Li_2_S from 2.842/2.261 to 2.384/1.441 eV (Fig. 2e, f). Besides, the 1 T phase of Co-MoS_2_ was increased accompanied by the decrease in sulfur defect when the Co content increased, further improving the adsorption and the electron transfer for LiPSs conversion [45]. Cation doping can facilitate the reaction kinetic for both discharge process and charge process. Benefitting from the enhanced catalytic effect, Co-doped SnS_2_ could promote the conversion of LiPSs to Li_2_S and reverse oxidation of Li_2_S. This was amply demonstrated by the obvious difference of S K-edge XANES, in which the feature of S–S disappeared during discharging from 2.0 to 1.7 V and re-appeared during charging process for S/NCNT@Co-SnS_2_ [46].

Doping modification may introduce lattice distortion, which can enrich the active sites and regulate the electron structure to enhance the catalytic activity. Xu-doped CoTe_2_ with Zn (Co_0.9_Zn_0.1_Te_2_) and introduced lattice strain, which changed the coordination environment of Co atoms and further reduced the *d*-band center. Because the adsorption strength is related to the *d*-band center, and too strong adsorption is not conducive to the further conversion of LiPSs, the decline of* d*-band center of Co_0.9_Zn_0.1_Te_2_ compared to CoTe_2_ meant more electrons occupied in the antibonding orbitals, which was conducive to the desorption of LiPSs to preserve its effective catalytic active site. Furthermore, the charge number of Te atom near Zn atom was also increased, which enhanced the affinity between Te and Li, improving the anchoring capacity for LiPSs. As shown in Fig. 2g, h, the lattice strain enhanced the intrinsic catalytic activity, promoting the break of bonds of LiPSs/Li_2_S, thus reducing the energy barrier of Li_2_S_4_ → Li_2_S_2_ → Li_2_S from 2.05 and 1.94 eV for CoTe_2_ to 0.51 and 1.6 eV for Co_0.9_Zn_0.1_Te_2_ [47]. Chen dissolved V into TiN lattice and formed solid solution Ti–V–N (TVN) in which V acted as dopant. As a result, Ti-N bonds were shorted while V–N bonds were lengthened, and the lattice parameter of TVN was reduced for the smaller atomic radius of V. More importantly, the structural distortion led to the *d*-band center of Ti lower while that of V increased, rendering V more effective to adsorb and catalyze LiPSs conversion. The best regulation was achieved at the Ti/V ratio of 4 for the largest structural distortion and highest *d*-band center of V, endowing the corresponding LSB with a retained capacity of 1036.8 mAh g^−1^ and a high capacity retention of 97.7% at 0.2 A g^−1^ for 400 cycles [48].

**Fig. 3:**
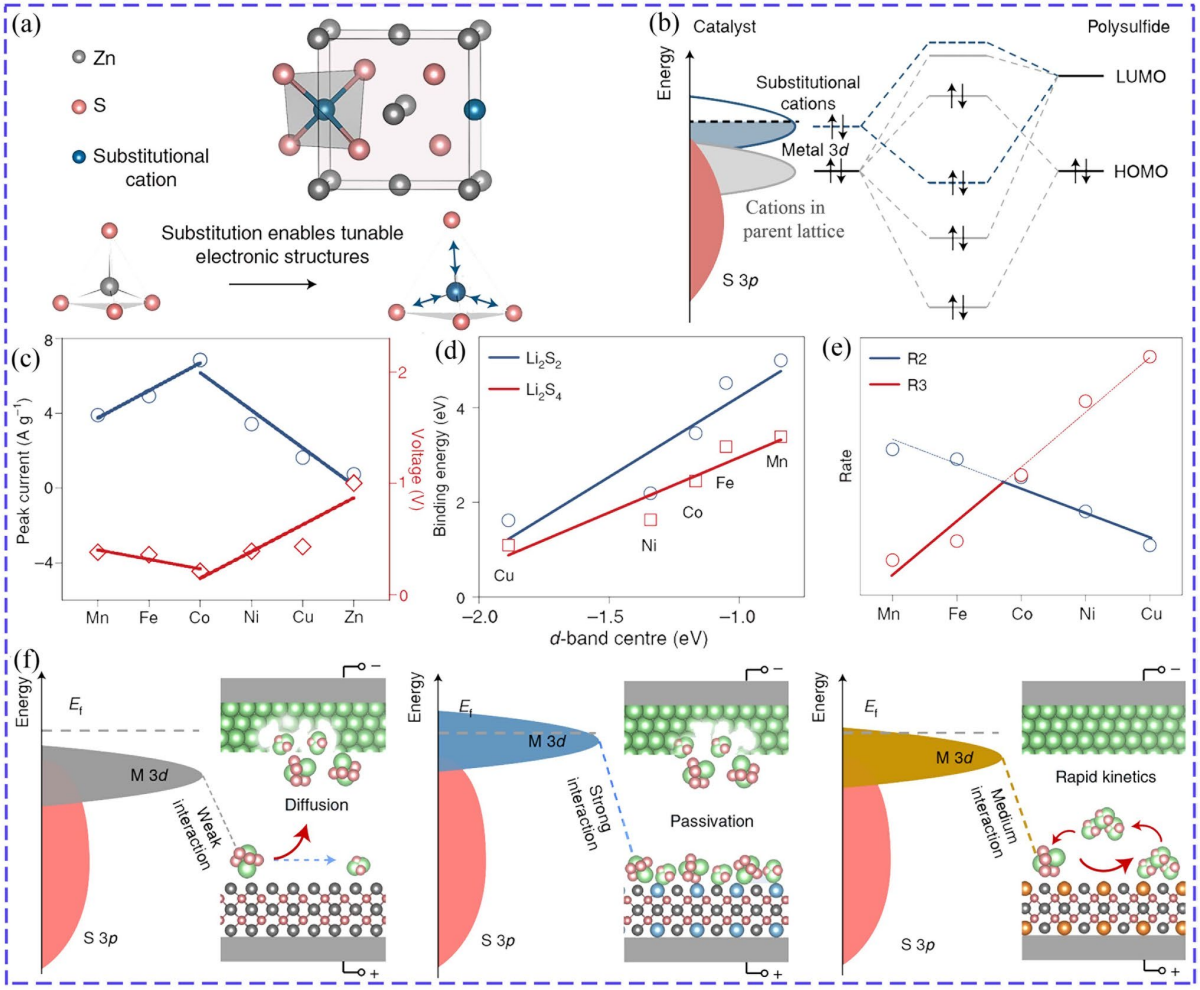
Relationship between catalytic activity and adsorption ability: **a** Schematic of doped ZnS by substituting Zn to tune the electronic structures. **b** Principle of regulating electronic structure by doping. **c** Voltage difference and peak current of the CV of symmetric cells. **d** Relationship of binding energies and *d*-band center. **e** Volcano diagram of reaction rates for ZnS doped with different ions (R2: LiPSs dissociation, R3: desorption steps). **f** Relationship of interactions and catalytic effect [51] Copyright: 2022, Springer Nature

Of course, the catalytic effect is also heavily affected by the type of doping ions. Selecting suitable doping ions is of great significance to optimize the catalytic performance by tuning electronic structures (Fig. 3a), especially the *d*-band due to its electronic interaction with LiPSs (Fig. 3b), which also closely relates to adsorption strength. Zhao et al. doped SnSe with kinds of transition metals Ti, V, Mn, Fe, Co, Ni, Cu, Zn, and proved that all the doping significantly improved the adsorption ability toward LiPSs/S_8_, and promoted LiPSs/S_8_ conversion based on Gibbs free energy results. However, not all doping could reduce the length of Sn–S bond formed between SnSe and LiPSs/S_8_, indicating different catalytic mechanisms [49]. Wang studied the influence of the electron structure on adsorption ability and catalytic performance of SmMn_2_O_5_, which was doped with major-group metals Mg, Ga and transition metals V, Cr, Fe, Mo at Mn site. The results showed that the binding energy for Li_2_S_4_, which was controlled by charge transfer, electronegativity difference between doped metal and S as well as surface work function of catalysts, had a linear relationship with the overpotential of sulfur reduction. Additionally, doping with Mg and Ga upward shifted the *d*-band center, while doping with transition metal had little effect [50]. Zhang incorporated metal ions (Mn^2+^, Fe^2+^, Co^2+^, Ni^2+^ and Cu^2+^) into ZnS to study the relationship of catalytic activity and adsorption ability. As a result, the catalytic ability showed a volcano-shaped trend from Mn to Cu-doped ZnS based on the peak current of CV of symmetric cells (Fig. 3c). The adsorption ability of doped ZnS exhibited a decreased trend from Mn to Cu with downshift of *d*-band centers (Fig. 3d), while a volcano plot of reaction rates of LiPSs dissociation and desorption steps was obtained (Fig. 3e). This was because that the catalytic activity increased with improved adsorption ability, while too strong adhesion of Li_2_S passivated catalysts (Fig. 3f). Consequently, the catalytic activity showed a volcano-shaped relationship with adsorption ability, and Co-doped ZnS with medium adsorption ability achieved the best catalytic effect [51].

The properties of doping ions that boost the catalytic activity of the materials have also been studied. For promoting LiPSs adsorption and electron exchange, Mn, Fe, Co, Ni with more *d* electron numbers than V were doped into VN to adjust the electronic structure of VN by enriching its *d* electrons. As a result, adsorption energy (5.86 eV for Li_2_S_4_) and interface electrons transfer (0.32 e) between LiPSs and catalysts were enhanced as Co was doped into VN in contrast to undoped VN (3.52 eV), which was ascribed to the lower filling fraction and higher *d*-band center of V 3*d*-band compared with undoped VN. Moreover, the best cathode performance could be obtained by Co-doped VN with retained capacity of 447 mAh g^−1^ at 3C for 500 cycles, which was more stable than other doped samples [52]. Regulating the electronic structure of VN with *d* electron-rich elements (Mn, Fe, Co, Ni) could indeed improve the LiPSs adsorption and redox reactions. However, the relationship between the number of *d* electrons and catalytic performance is not concluded.

The electron donating ability of dopant ions also plays a key role in improving catalytic activity [53]. Li proposed Cu as electron donor to dope CoP/MXene and control the redox kinetics of Li_2_S by regulating its electron structure and enriching active sites. To be specific, the doped Cu rendered the Co atoms charge accumulation, introducing unbalanced charge distribution and forming more Co/Cu–S bonds, which eventually increased the binding energy from 2.43 to 5.58 eV (Cu_0.1_Co_0.9_P), as shown in Fig. 2i. On the other hand, the strongly electronegative Co^3+^ (15.26) in CoP was transformed into weakly electronegative Co^2+^ (9.10) by trapping electrons from the Cu atoms. This weakened the Co–S bond energy and lengthened the bond from 2.150 to 2.168 Å (Fig. 2j), while producing much lattice vacancy. As a result, the diffusion energy barrier and activation energy of nucleation/decomposition of Li_2_S were reduced (Fig. 2k), improving the redox kinetics of Li_2_S. With accelerated redox kinetics and enhanced sulfur utilization, the sulfur cathode with Cu_0.1_Co_0.9_P could achieve a specific capacity of 1475 mAh g^−1^ at 0.2C and a retention of 73.4% for 100 cycles, superior than that of CoP (57.6%) [54].

In addition, as the electronegativity affects the ability of TMCs to attract bonding electrons, the binding strength and electron transfer between TMCs and LiPSs are also affected [55]. Therefore, electronegativity is also a key factor of doped ions to improve the catalytic performance. Hu-doped NiSe_2_ with Fe with lower electronegativity than Ni and acted as electron donor to enhance the electron transfers from Fe-NiSe_2_ to LiPSs. As a result, Fe-NiSe_2_ improved chemisorption ability with the formed S-Fe and shortened S-Ni and Li-Se bonds between Fe-NiSe_2_ and Li_2_S_6_. Additionally, Fe doping resulted in the increase in density of states (DOS) near the Fermi level and antibonding orbitals in conduction band, indicating improved electrical conductivity, which was conducive to catalyzing the redox of sulfur species [41]. CoB was doped with Mo with higher electronegativity than Co, forming a metal compound structure, and causing detachment of B and insertion/extraction of Li^+^ to the vacancy during discharging and charging. Co_7_Mo_3_B was more prone to anchor long-chain LiPSs with higher binding energies and simultaneously conducive to the dissolution of Li_2_S_2_/Li_2_S with lower binging energy than CoB. As a result, the interaction of Co, B and Mo atoms could bidirectionally promote the redox kinetics [56]. In addition, anti-selfdischarge behavior, ionic conductivity and Li^+^ transportation could also be improved by doping modification, such as Ni-doped WS_2_ [40].

The doping amount is also an important factor modifying catalysts. For example, when Fe doping content of TiO_2_ increased from 1 to 5%, the capacity decay rate decreased from 0.27 to 0.08% at 1C for 500 cycles with accelerated kinetic reaction and decreased electrode polarization [57]. Zhang et al. doped Fe into Co_3_O_4_ and formed Co_3_O_4_ hollow spheres with multi-shell structure and oxygen defects by adjusting the Fe doping amount. As a result, the electronic conductivity of Fe/Co_3_O_4_ and the chemical adsorption for LiPSs were significantly improved, and catalytic sites were enriched, promoting the LiPSs conversion [58].

In addition to type and content of doping ions, doping site is also a key factor affecting the doping effect. Mn, Fe, Co, Ni etc. were selected to dope MoS_2_ at Mo and S sites, respectively. Doping MoS_2_ at Mo sites could not obviously enhance the binding strength for LiPSs. In contrast, doping at S sites improved the binding energies due to more electrons transferred from the adsorbed LiPSs to doped MoS_2_, which was ascribed to much stronger orbital overlap of Co-3*d* and S-3*p* of Li_2_S_4_ taking Co-doped MoS_2_ as an example. Furthermore, doped MoS_2_ by substituting S exhibited good catalytic activity for LiPSs conversion with low Gibbs free energies [59]. Doping site also has obvious influence on the band structure. Band structure of different types of Ti-doped SiO_2_ were calculated, including substitutional type (Ti(S)-SiO_2_) and impurities types which connected one O atom (Ti(I_1_)-SiO_2_), two O (Ti(I_2_)-SiO_2_) and O/Si (Ti(I_3_)-SiO_2_). Their band gaps were 4.528, 2.468, 0.483 and 0.955 eV, respectively, which were smaller than that of pure SiO_2_ (5.672 eV) [60].

#### Anion Single-Doping Modification

**Fig. 4:**
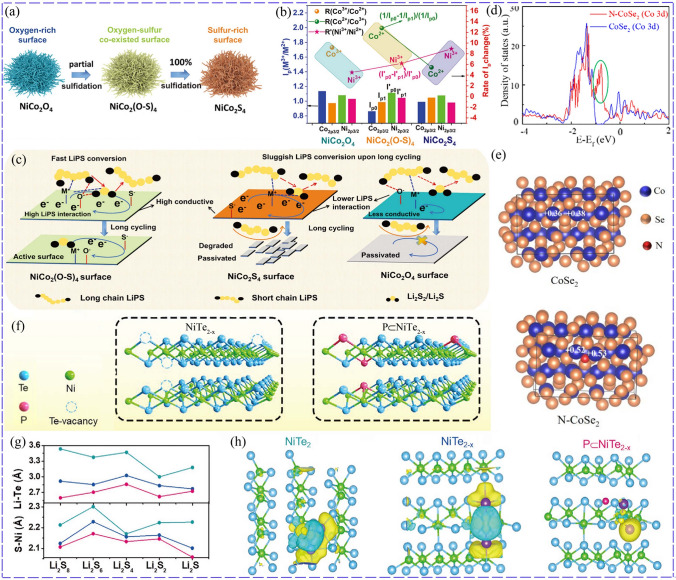
Superior effect of O-doped NiCo_2_(O–S)_4_: **a** sulfidation process NiCo_2_(O–S)_4_ and NiCo_2_S_4_. **b** Ratio of M^3+^/M^2+^ 2p_3/2_ peak intensity (I_p_) and the rate of I_p_ change. **c** NiCo_2_(O-S)_4_ with high conductivity and LiPSs affinity greatly reserved the catalysis active surface [62]. Copyright: 2022, John Wiley and Sons. Improvement of catalytic effect by N-doped CoSe_2_: **d** PDOS of CoSe_2_ and N–CoSe_2_. **e** Charge number of Co in CoSe_2_ and N–CoSe_2_ [63]. Copyright: 2020, American Chemical Society. P-doped NiTe_2−*x*_: **f** Schematic illustration of P-doped NiTe_2−*x*_ at Te vacancies. **g** Difference of Li–Te and S–Ni bond lengths formed between LiPSs and various catalysts. **h** Charge density difference between Li_2_S and catalysts, the yellow (blue) distribution corresponds to charge accumulation (depletion) [65]. Copyright: 2022, John Wiley and Sons

Anions doping can also promote the catalytic activity of TMCs with modified electronic structure, such as the *d*-band center and electron filling of metal ions. Firstly, anions doping can also enhance the electronic conductivity, such as S-doped Co_0.85_Se (1.05 eV) with lower band gap compared with that of Co_0.85_Se (1.07 eV). Moreover, the growth of Li_2_S could be regulated via the synergistic adsorption by Se and S [61]. Anions doping can also enhance the chemical interaction with LiPSs. Wu-doped NiCo_2_S_4_ with high amount of oxygen (37.28%) (Fig. 4a), obtaining NiCo_2_(O–S)_4_ with lower conductivity (30.1 S cm^−1^) but superior trapping ability for LiPSs than natively oxidized NiCo_2_S_4_ with O content of less than 10.0% (51.2 S cm^−1^). Specifically, Co was the dominated LiPSs interaction site of NiCo_2_O_4_ and NiCo_2_(O-S)_4_, while that of NiCo_2_S_4_ was Ni, which was proved by the intensity changes of Ni/Co XPS peaks before and after interacting with LiPSs (Fig. 4b). Electron transferred from the Li_2_S_6_ to the O atoms, forming Li–O–M. As a result, NiCo_2_(O-S)_4_ presented the best catalytic effect for promoting the LiPSs conversion than that of NiCo_2_O_4_ with high charge transfer barrier due to poor conductivity and NiCo_2_S_4_ for weaker interaction with LiPSs (Fig. 4c). As expected, the LSB of NiCo_2_(O–S)_4_ achieved a preferable cycling stability compared with NiCo_2_O_4_ and NiCo_2_S_4_, maintaining a capacity of 962 mAh g^−1^ after 200 cycles at 0.2C and 922 mAh g^−1^ after 150 cycles at 0.5C [62].

The properties of doped anions and promoted mechanism that enhance the catalytic activity of TMCs have also received attention. N with higher electronegativity than Se was selected to modify CoSe_2_, which could elongate S–S of LiPSs and Li–S bond of Li_2_S but shorten Co-S bond, resulting in facilitated conversion of sulfur species both in discharge and charge process. The enhanced chemisorption and catalytic capacity of N-CoSe_2_ was due to less filled antibonding states and facilitated charge transfer with higher *d*-band center of Co compared to CoSe_2_ (Fig. 4d). In addition, the charge number of Co atoms was increased to provide more empty orbitals for adsorbing LiPSs (Fig. 4e) [63]. N doping could also tune the electron filling state of TMCs to regulate the adsorption ability as well as conversion kinetics for LiPSs. The electron density of Ta around N in N-Ta_2_O_5_ was increased compared to Ta_2_O_5_, because fewer electrons in Ta *d* orbitals flowed into N than O for the relatively weak electron binding ability of N. As a result, *d* orbitals electron filling of Ta in N-Ta_2_O_5_ (2.96) was higher than that of Ta_2_O_5_ (2.87) but lower than that of Ta_3_N_5_ (3.23), corresponding to the charge transfer amount between Ta of N-Ta_2_O_5_, Ta_2_O_5_, Ta_3_N_5_ and S, which imparted N-Ta_2_O_5_ (3.70 eV for Li_2_S_4_) moderate ability to anchor LiPSs compared to that of Ta_2_O_5_ (6.94 eV) and Ta_3_N_5_ (2.82 eV). The sulfur cathode with N-Ta_2_O_5_ exhibited the highest initial capacity of 1252.8 mAh g^−1^ and capacity retention rate of 92.7% at 0.2C for 100 cycles among the studies catalysts [64].

P with higher electronegativity than Te was also selected to dope NiTe_2_ with Te vacancies. The P atoms occupied Te vacancies instead of taking the place of Te atoms (Fig. 4f). The higher electronegativity of P than that of Ni and Te rendered it attract nearby atoms and trigger bonds reconstruction, shortening the Ni-Te of P-NiTe_2-*x*_. Additionally, the shortest S-Ni and Li-Te bonds between P-NiTe_2-*x*_ and LiPSs (Fig. 4g) demonstrates the strongest anchoring effect. And electron density of Ni-S was decreased while that of Li-Te was increased when P-NiTe_2−*x*_ interacted with Li_2_S (Fig. 4h), meaning a promoted catalytic activity [65]. However, Hu et al. doped P with lower electronegativity than Se into NiSe_2_. As a result, the electron-rich P promoted the electrons transfer from Li_2_S_6_ to P-NiSe_2_, and the S at the end of Li_2_S_6_ showed apparent electron deletion, rendering it present superior catalytic effect than NiSe_2_ [66]. *P* was also used to dope MoS_2_ and hybridized with Mo 3*d* and S 2*p* orbital, resulting in more charge transfer and superior conductivity. This induced stronger Mo–S and P–Li bonds and longer S–S/Li–S bonds of LiPSs/Li_2_S to accelerate their conversion [67]. Similarly, CoS_2_ doped with P with relatively low electronegativity also improved the catalytic activity [68]. Therefore, whether the electronegativity of doping ions is the key factor to regulate the catalytic activity of TMCs, and their relationship as well as the promoted mechanism needs to be further studied.

The valence states of TMCs can also be optimized by doping to enhance catalytic activity. Chen’s group modulated the valence states of Ni and Zn for Ni_3_ZnC_0.7_ by P doping and accompanied Zn vacancies, which remarkably decreased the electron density at Zn sites to anchor LiPSs. Meanwhile, the content of Ni^2+^ species was slightly increased to facilitate electron transfer between Ni^2+^/Ni(0) and LiPSs, improving the catalytic effect for LiPSs conversion. The LSB with P-doped Ni_3_ZnC_0.7_ modified separator could present a more stable cycle performance with an initial capacity of 684 mAh g^−1^ and fading rate of 0.0247% at 1C for 1400 cycles, in contrast to that of pristine Ni_3_ZnC_0.7_ with 583 mAh g^−1^ and 0.0341%, respectively [42].

#### Dual-Doping Modification

**Fig. 5:**
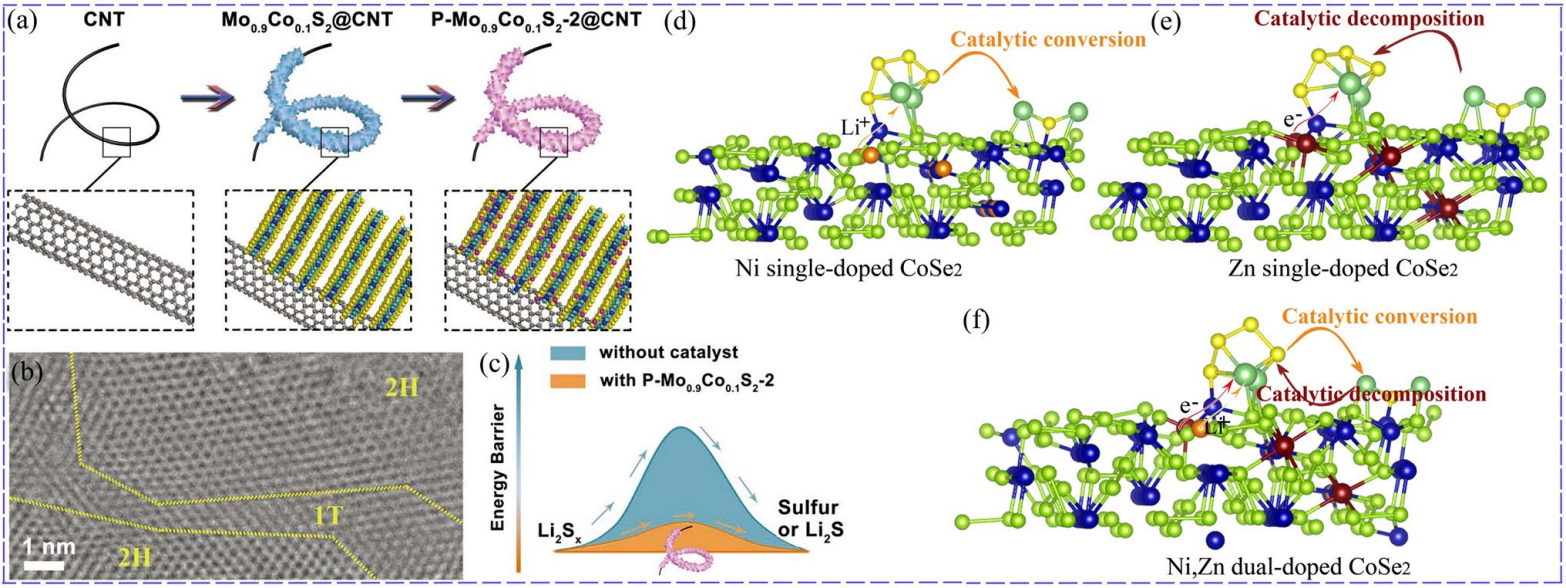
Co, P co-doped MoS_2_: **a** Schematic illustration of Co, P co-doped MoS_2_ (P-Mo_0.9_Co_0.1_S_2_). **b** HRTEM images of Co, P co-doped MoS_2_. **c** Energy barrier of LiPSs conversion [69]. Copyright: 2019, John Wiley and Sons. Schematic illustration of the functions of different catalysts: **d** Ni-doped CoSe_2_, **e** Zn-doped CoSe_2_, and **f** Ni/Zn-doped CoSe_2_ [55]. Copyright: 2021, John Wiley and Sons

Due to the synergistic effect between doping ions, dual-doping modification could obtain better catalytic performance than single doping. Lee et al. designed Co and P dual-doped MoS_2_ (Fig. 5a), in which Co doping changed MoS_2_ phase from 2H to 1 T, improving its conductivity and providing more effective electron conduction (Fig. 5b). More importantly, the Co–P coordination formed after P doping further improved the catalytic activity of Mo_0.9_Co_0.1_S_2_. As a result, Co and P co-doping improved the catalytic performance for LiPSs conversion with lower energy barriers (Fig. 5c). Benefitting from the synergistic effect of Co and P dual-doping, P-Mo_0.9_Co_0.1_S_2_ obtained the best cathode performance. Represented by the rate performance, P-Mo_0.9_Co_0.1_S_2_ presented a high capacity of 931 mAh g^−1^ at 6C, in contrast to that of Mo_0.9_Co_0.1_S_2_ with 633 mAh g^−1^ and MoS_2_ with 338 mAh g^−1^ [69]. Li et al. modified CoSe_2_ with Ni and Zn dual-doping to bidirectionally catalyze the redox of sulfur cathodes. To be specific, the catalytic effect of Ni-doped CoSe_2_ for the LiPSs conversion in the discharge process was better than that of Zn doping modification, while Zn doping achieved better catalytic performance on the Li_2_S decomposition. In addition, Ni/Zn dual-doping CoSe_2_ had the better catalytic performance than that of the mixture of Ni-doped CoSe_2_ and Zn-doped CoSe_2_ [55]. TMCs co-doped by anions was also involved. N, F, and B were co-doped into CoFe_2_O_4_ and induced rich O vacancies to enhance the conductivity and act as adsorption, catalytic sites for LiPSs. The density functional theory (DFT) results also showed that new electron energy peaks appeared and increased the carrier density after the anions co-doping modification. Consequently, the improved binding energy and catalytic effect of modified CoFe_2_O_4_ suppressed the shuttle effect and boosted the redox of sulfur cathodes, delivering an excellent electrochemical performance with maintained capacity of 1156 mAh g^−1^ at 0.2C for 300 cycles as separator coating layer, which exceeded that of pristine one with only 825 mAh g^−1^ [70].

Metal ion-doped metal compounds are usually prepared in two ways. The first is to directly add stoichiometric-doped metal salt during the hydrothermal reaction or co-precipitation reaction. For example, Ni-doped MoS_2_ was prepared by hydrothermal treatment of the mixture of oxalic acid, thiourea, (NH_4_)_6_Mo_7_O_24_·4H_2_O and Ni(Ac)_2_·4H_2_O, and the doping content could be controlled by amount of metal salts [43]. The second is adding the metal salt of the doping ions to some solvent along with the pristine metal compound or its precursor, and further replace the metal ions in the metal compound for doping through heating or hydrothermal conditions. Taking doped SiO_2_ as an example, M-SiO_2_ (M = Ti, Al, Sn) were synthesized by hydrothermal treating the mixture of SiO_2_ and stoichiometric metal source in isopropyl alcohol [60]. As for anion doping of TMCs, metal compounds and chemical reagents containing doping sources are usually calcined at high temperatures, such as ammonium hydrogen carbonate, sodium hypophosphite and sodium hydrogen sulfite as a nitrogen source, phosphorus source and sulfur source, respectively. For example, N-doped CoSe_2_ was synthesized by calcining the CoSe_2_ accompanied with ammonium hydrogen carbonate [63]. Furthermore, anion and cation co-doping can be carried out by single doping of metal ion and then anion doping.

Although reasonably engineering TMCs catalysts could promote the LiPSs conversion, the design and regulation of ideal bidirectional sulfur catalyst is still a great challenge for the difficulty of selecting ions with different functions [71]. Different doping ions in TMCs may play various functions, such as improving conductivity, regulating electronic structure, and adsorbing-catalyzing different LiPSs in different stages of LSBs. Moreover, multicomponent co-doping may enrich the active sites and play a synergistically catalytic role [72]. However, research on this aspect is lacking, and it is worth further study, especially the selection and matching of multi-doped ions and their synergistic catalysis mechanism through deeply studying the regulation rules of electronic structure. Based on this development, high-entropy doping may be proposed. Additionally, it is found that the external magnetic field could regulate the spin polarization of CoS_*x*_ catalyst, drive the transition of Co^3+^ electrons from the low spin state to the high spin state, generating additional unpaired electrons in the 3*d* orbit of Co and enhancing the hybridization between Co-3*d* and S-2*p* orbital. As a result, the charge transfer dynamics at the CoS_*x*_/LiPSs interface was promoted, and the adsorption-catalytic capacity for LiPSs was significantly improved [73]. Doping is also an effective strategy to manipulate spin polarization of catalysts to enhance the catalytic activity [74, 75]. Therefore, the adjustment of spin effect induced by doping should be paid enough attention in LSBs. In addition, doping modification may introduce defects such as vacancy in TMCs, which may provide additional adsorption and activation sites [76]. It is worth noting that doping may cause changes in *d*-band center, spin polarization, lattice distortion and vacancy defects at the same time. Therefore, controlling electronic structure through doping is complicated and these factors should be comprehensively investigated. Furthermore, directed doping to specific sites should be developed for higher catalytic activity [77].

### Multi-Ionic TMCs

Multi-ionic TMCs composed of more metal ions or anions also possess enriched active sites, optimized chemical properties, and improved catalytic activity compared with TMCs of one component. In contrast to doping modification which belongs to a kind of defect and retain the phase and crystal structure of the original TMCs, bimetal TMCs with fixed atomic ratios within a certain range are solid solutions and possess unique crystal structures, such as Co_3_Mo_3_N, BaTiO_3_ and FeWO_4_, and only some may be identical to one-component TMCs, such as Ni_2_Co_4_P_3_. Furthermore, doping modification can be achieved by introducing heterogeneous ions, but the synthesis methods cannot be simply transplanted to the preparation of dual-ionic metal compounds. Multi-ionic TMCs are usually not obtained by increasing the content of doped ions in TMCs. Increasing the doping amount may lead to changes in crystal phase besides changed lattice parameter and reconstructed bonds, and even anion aggregation and phase collapse [78, 79].

#### Multi-Metallic TMCs

##### Bimetallic TMCs

**Fig. 6:**
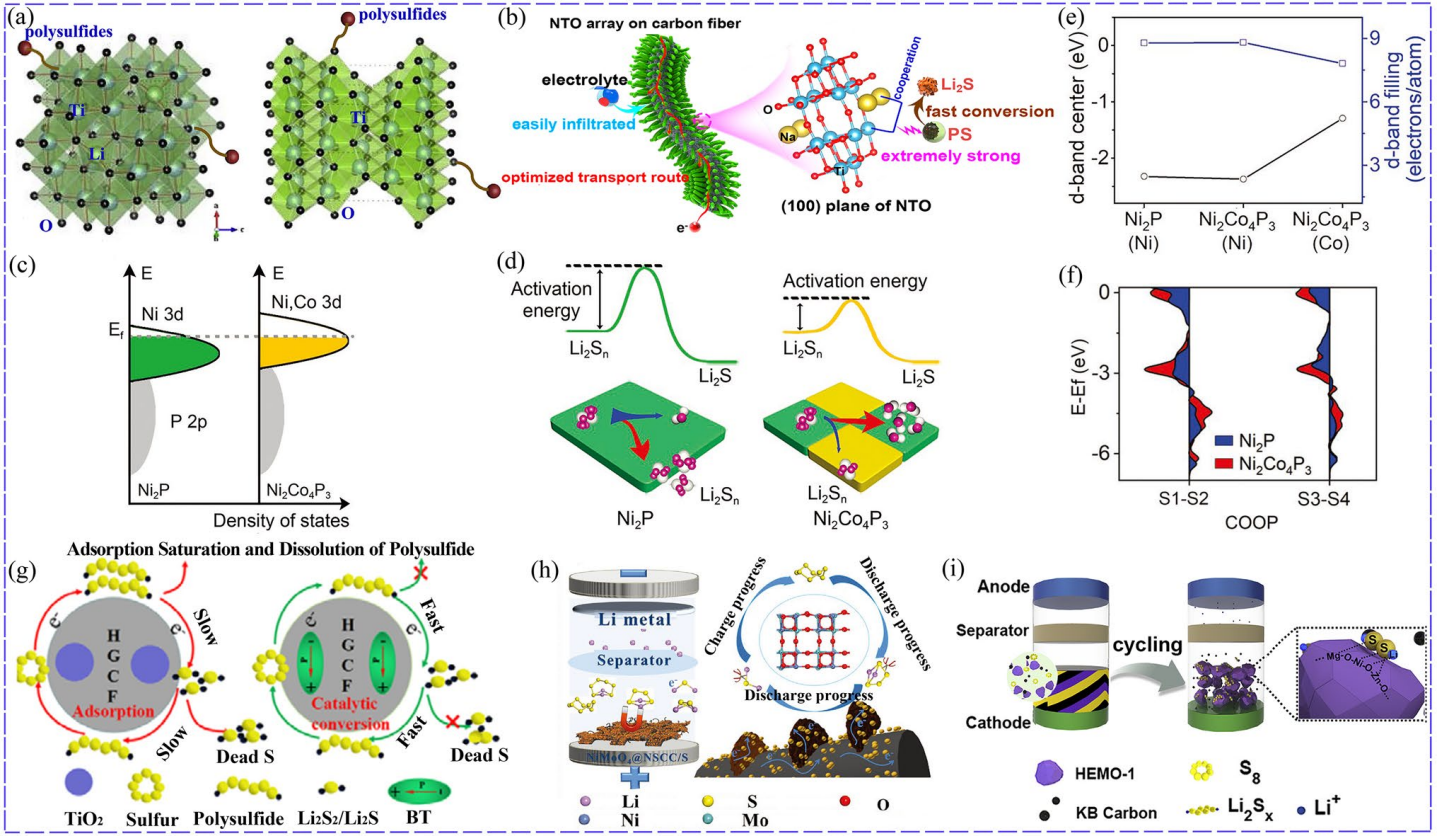
**a** Different bonds formed between Li_4_Ti_5_O_12_, TiO_2_ and LiPSs [83]. Copyright: 2019, Elsevier. **b** Advantages of Na_2_Ti_6_O_13_ array [84]. Copyright: 2021, American Chemical Society. **c** DOS of Ni/Co phosphides. **d** Activation energy of Li_2_S nucleation on Ni_2_P and Ni_2_Co_4_P_3_. **e** *d*-band center of Ni_2_P and Ni_2_Co_4_P_3_. **f** COOP diagram of the S1-S2 and S3-S4 bonds of Li_2_S_4_ [90]. Copyright: John Wiley and Sons. **g** Schematic diagram of TiO_2_ as adsorption material while BaTiO_3_ (BT) as electrocatalyst [91]. Copyright: 2020, Elsevier. **h** Functions of NiMoO_4_ for LiPSs [98]. Copyright: 2021, John Wiley and Sons. **i** Schematic diagram of high-entropy metal oxide (HEMO) for adsorbing LiPSs [110]. Copyright: 2019, Elsevier

Compared with corresponding single-component compounds, bimetallic TMCs can achieve better conductivity and electrochemical activity, which have also been applied to catalyze LiPSs conversion. Zhang synthesized NiCo_2_S_4_ microspheres, which possessed good electrical conductivity and catalytic activity, ensuring fast conversion kinetics of LiPSs [80]. Ni-Co phosphide nanoparticles embedded in carbon hollow nanocages were also designed as adsorption-catalytic sites, promoting redox reaction and Li^+^ diffusion in LSBs [81]. The reasons why bimetallic TMCs are superior to monometallic TMCs have also been studied. NiCoP possessed a significantly higher binding energy of 4.24 eV for LiPSs than CoP (1.48 eV), because the natural oxidation of NiCoP formed Ni–O–P and Co–O–P and activated the Co/Ni sites, rendering it bind with LiPSs through Co-S and Ni-S bonds [82]. In addition, bimetallic TMCs can also establish more suitable chemical bonds to optimize chemisorption effect. For example, TiO_2_ anchored LiPSs with S–O bonds, while bimetal Li_4_Ti_5_O_12_ could form more efficient Ti–S bonds (Fig. 6a), resulting in excellent cyclic stability of sulfur cathode at sulfur loading of 4 mg cm^−2^ with 80% capacity retention at 1C for 300 cycles [83]. Na_2_Ti_6_O_13_ with synergistic coordination of Na and Ti provided double-cations as adsorption sites for promoting LiPSs conversion and preventing their accumulation (Fig. 6b). Furthermore, the distortion of TiO_6_ octahedron in Na_2_Ti_6_O_13_ generated a large dipole moment, and the internal polarization field in TiO_6_ octahedron was favorable for electron transfer, promoting the conversion of LiPSs and significantly enhancing the adsorption ability compared with TiO_2_. The LSB with Na_2_Ti_6_O_13_ could reach a capacity of 815 mAh g^−1^ at 1C and maintained its capacity at 84% at 0.1C for 100 cycles compared with that of TiO_2_ at 75% [84]. Additionally, the conductivity and charge transport could also be improved compared to the monometallic compounds [85].

The fundamental reasons for elevated adsorption and catalytic ability of bimetallic TMCs are further explored. Electron transfer between different metals of bimetallic TMCs can tune the electronic structure such as distribution of electron density, thus adjusting the adsorption and catalytic properties [86]. Zhang designed Co_3_Mo_3_N by calcinating Mo-doped ZIF-67, and the electron redistribution was occurred with the introduction of Mo due to its greater electronegativity than Co. As a result, electron transferred from Co and Mo to N atoms, and the electron density around Co was decreased while that of N was increased, facilitating the adsorption and conversion of LiPSs [20, 87].

In addition, tuned valence state is also an important reason for enhanced catalytic capacity of bimetallic TMCs. For example, electron transfer was occurred from Co to Sn in CoSn(OH)_6_ due to their different electronegativity, which increased the Co^3+^ and decreased the valence state of Sn compared with Co(OH)_2_ and Sn(OH)_4_. As a result, the chemical interaction between Co and S were enhanced, while that of Sn and S was weakened for CoSn(OH)_6_ after discharge–charge, presenting a moderate adsorption capacity, which promoted the LiPSs diffusion for conversion [86]. Chemical valence could also be varied after interaction with S/LiPSs to boost the redox of sulfur species. Co^3+^ and Ni^3+^ were in situ formed attributed to the interaction between nickel–cobalt double hydroxide (NiCo-DH) and sulfur, which was conducive to the oxidation of initially formed LiPSs and the formation of thiosulfates. The reversible redox between thiosulfates and LiPSs could confine LiPSs and catalyze their conversion. Moreover, NiCo-DH underwent a redox with Li_2_S_6_, partially reducing Co^3+^ and Ni^3+^ to Co^2+^ and Ni^2+^. The electron transfer and strong coupling effects between Ni and Co in NiCo-DH rendered it a smaller redox potential [88]. Li_*x*_MoO_*y*_ was in situ synthesized by electrochemically activating MoO_3_ at about 2.6 V, which subjected to a conversion between Li_0.042_MoO_3_ and Li_2_MoO_4_ at about 2.2 V, thus accelerating the redox of LiPSs with the potential overlap [89].

Interaction between metal sites can also regulate the *d*-band electron filling and *d*-band center, thus improving chemisorption and catalytic property for LiPSs. Zhang alloyed Co to Ni_2_P to improve the *d*-band of Ni_2_Co_4_P_3_ (Fig. 6c), in which the Co 3*d*-band in Ni_2_Co_4_P_3_ was higher than the Ni 3*d*-band in Ni_2_Co_4_P_3_ and Ni_2_P. Because Co 3*d* had lower electron fill number and higher *d*-band center than Ni 3d (Fig. 6e), Ni_2_Co_4_P_3_ was endowed with enhanced interaction with LiPSs and reduced activation energy for LiPSs conversion (Fig. 6d). Additionally, Ni_2_Co_4_P_3_ formed a shorter metal-S bond with LiPSs and weakened S–S bonds of LiPSs, reducing the energy consumption of S–S bonds and energy barrier for conversion reaction compared with Ni_2_P. The crystal orbital overlap population of S–S bond at both ends showed that the electron filling of antibonding state of Li_2_S_4_ on Ni_2_Co_4_P_3_ was higher than Ni_2_P (Fig. 6f) [90].

For host materials with relatively low conductivity but strong interaction with LiPSs, such as metal oxides, the surface diffusion of LiPSs/Li_2_S is vital for electrochemical reactions [19]. Fortunately, bimetallic TMCs can also promote the diffusion of Li_2_S. For example, BaTiO_3_ delivered a much lower diffusion energy barrier of Li_2_S than TiO_2_, showing superior surface diffusion kinetics and more uniform dispersion of Li_2_S. More importantly, BaTiO_3_ with inherent self-polarization showed promoted redox kinetics of LiPSs/Li_2_S than TiO_2_ (Fig. 9g). As a result, the LSB equipped with BaTiO_3_ could deliver an initial capacity of 896 mAh g^−1^ with remained capacity of 466.1 mAh g^−1^ at 1C for 1000 cycles, exceeding that of TiO_2_ with retained capacity of 210 mAh g^−1^ [91]. Similarly, Bi_4_Ti_3_O_12_ with inner electric field induced by spontaneous polarization showed excellent catalytic activity for promoting LiPSs conversion [92]. The intermolecular polarization of CoIn_2_S_4_ and Li_2_S_4_ introduced by their strong interaction was regarded as an essential reason of reduced conversion barrier and enhanced charge transfer from CoIn_2_S_4_ to Li_2_S_4_ [93]. Additionally, Li^+^ diffusion can also be promoted. Compared to Ni_3_C, Ni_3_ZnC_0.7_ could provide Ni, Zn sites as sulfiphilic sites and lithiophilic sites, respectively, which reduced energy barriers of Li^+^ diffusion and improved catalytic property [94].

Bimetallic TMCs may also combine the advantages of the two metal ions or corresponding monometallic TMCs. Liu designed Co_3_V_2_O_8_ to make use of V, Co binary active sites to anchor LiPSs and catalyze their conversion [95]. FeWO_4_ nanorods was designed for combining the merit of Fe_2_O_3_ with strong affinity and WO_3_ with good catalytic property, delivering a faster Li^+^ diffusion rate, stronger chemical binding and better redox kinetics for LiPSs. Thanks to the simultaneous satisfaction of adsorption and catalysis, Li_2_S_6_ cathode with FeWO_4_ obtained more stable cycling properties with retained capacity of 519 mAh g^−1^ at current of 3.2 mA after 600 cycles than that of Fe_2_O_3_ only with 502 mAh g^−1^ after 150 cycles [96]. For MoWS catalyst, 1 T phase structure with better electron conductivity possessed higher catalytic activity, while 2H phase was beneficial for Li^+^ diffusion [87]. Mo_0.5_W_0.5_S_2_ composed of 2H-1 T mixed phase, possessed both superior catalytic activity for LiPSs conversion and a faster electron transport, delivering a better reaction kinetic than that of MoS_2_ and WS_2_ [97]. Bidirectional catalysts can also be constructed with bimetallic TMCs. For example, Shu introduced Mo into NiO and constructed NiMoO_4_ with increased electron density, which led to the decrease in bandgap from 2.51 eV (NiO) to 0.56 eV (NiMoO_4_) and the improvement of metallic properties. Furthermore, NiMoO_4_ rendered the S–S bonds and Li–S bonds of adsorbed Li_2_S_4_ and Li_2_S longer, and Gibbs free energy of the rate-determining step (Li_2_S_2_ → Li_2_S) and Li_2_S decomposition barrier for NiMoO_4_ (0.90 eV, 0.19 eV) reduced compared with NiO (1.15 eV, 1.73 eV), simultaneously facilitating the conversion of LiPSs and Li_2_S oxidation (Fig. 6h) [98].

Introducing metal ions may also affect the morphological structure to help optimize the cathode performance. Zhang prepared NiCo hydroxide polyhedrons by etching ZIF-67 with Ni(NO_3_)_2_·6H_2_O because of its lower pH than Co(NO_3_)_2_·6H_2_O in ethanol. The obtained NiCo hydroxide with denser and miniaturized nanosheets along the polyhedral shells, facilitated the exposure of enriched active surfaces for chemical anchoring LiPSs and catalyzing their reactions. Consequently, bimetallic NiCo hydroxide hollow polyhedra was more conducive to improving the electrochemical performance of LSBs, endowing the corresponding sulfur cathode with a higher capacity of 763 mAh g^−1^ after 100 cycles at 0.1C than that of Co hydroxide of only 406 mAh g^−1^ [99].

MOFs with the merits of tunable pore structures, structural diversity as well as functional versatility, are extremely suitable for chemical anchoring and physical confining LiPSs with Lewis acid–base interaction and porous structure, and catalyzing the LiPSs conversion [100, 101]. Furthermore, bimetallic MOFs can improve its electronic conductivity, enrich active sites, and plays synergistic effects. For example, considering that Al^3+^ in Al-MOF was coordinatively saturated and hardly to bind extra LiPSs, coordinatively unsaturated Cu^2+^ with sulfiphilicity was introduced to construct bimetallic Al/Cu-MOF, which generated additional sites for anchoring LiPSs and enhanced the interaction [102]. A Zr-Fc MOF possessed positively charged Zr sites provided by uncoordinated Zr-O defects and positively charged oxidized Fc^+^, to electrostatically adsorb LiPSs. At the same time, acidic protons in the defect sites of Zr-O nodes could also anchor LiPSs. These abundant anchor sites and electrocatalytic activity of Zr-Fc MOF enabled the LSBs a superior cycle stability compared to Zr-MOFs [103].

**Fig. 7:**
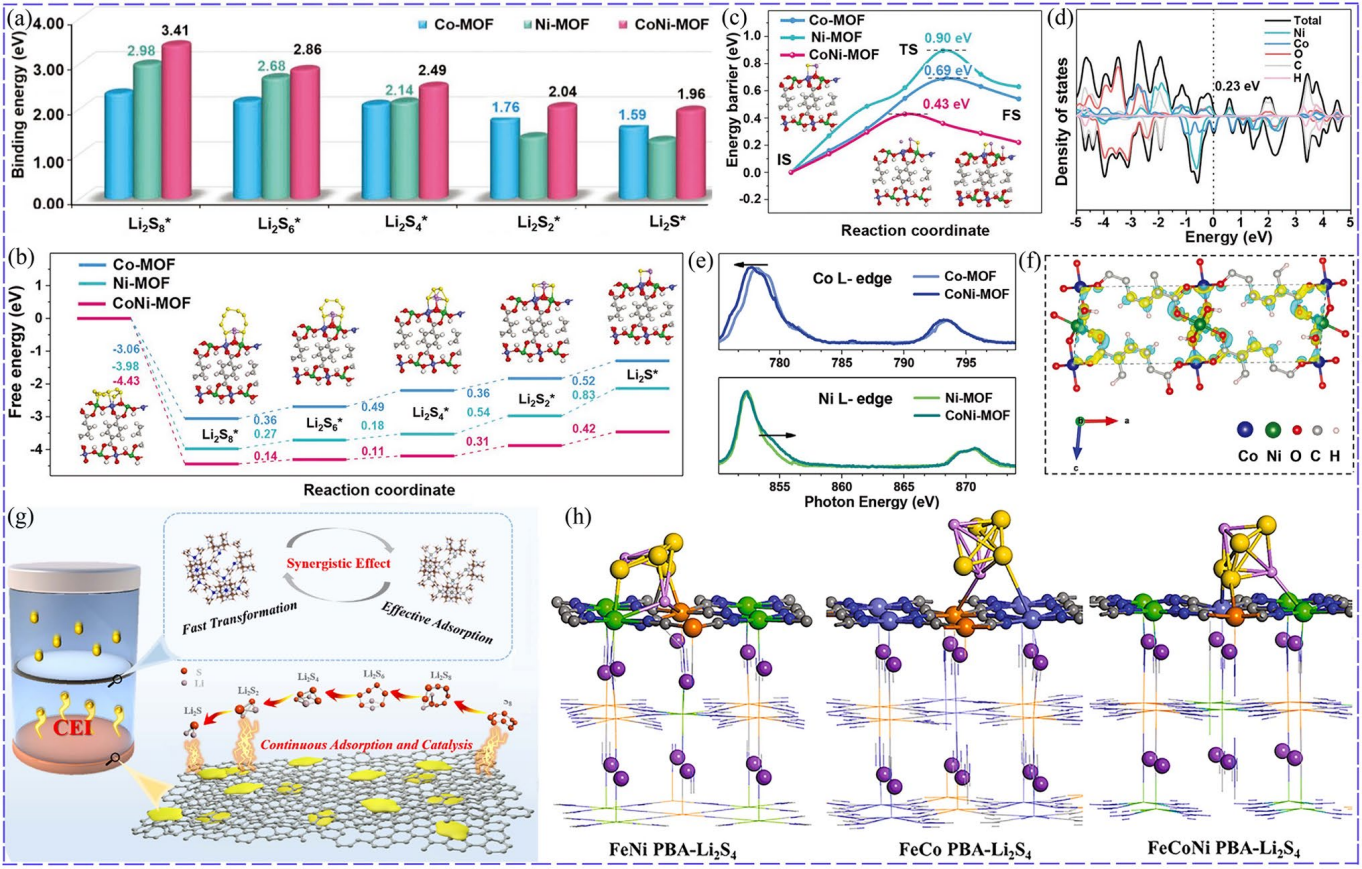
Synergistic catalysis of NiCo-MOF: **a** Binding energies. **b** Free energy for the discharging process. **c** Li_2_S decomposition energy barrier. **d** Calculated DOS. **e** Co L-edge and Ni L-edge XANES spectra of the MOFs. **f** Charge density difference of CoNi-MOF [104]. Copyright: 2021, John Wiley and Sons. **g** Synergistic effect of Zn and Co sites in bimetallic Zn/Co-ZIF MOF [12]. Copyright: 2022, Elsevier. **h** The strongest interaction obtained for FeCoNi-PBA with the increase in adsorption sites [113]. Copyright: 2021, Elsevier

To catalyze multi-step redox process, catalyst with two kinds of catalytic sites were proposed. NiCo-MOF was thus designed by combining the different catalytic function of Ni-MOF and Co-MOF. As shown in Fig. 7a-c, Ni-MOF preferred to bind the long LiPSs and delivered lower Gibbs free energy for their conversion, while Co-MOF anchored the short LiPSs stronger, facilitated their reduction and Li_2_S oxidation. Furthermore, NiCo-MOF possessed better conductivity (Fig. 7d) and improved catalytic activity, because charge redistributed between Ni and Co (Fig. 7e, f) for their asymmetric interaction with the bridge O, and led to the change of the unfilled metal electron orbitals [104]. Wang also designed NiCo-MOF and the charge transfer from Co to Ni due to the higher electronegativity of Ni^2+^ than Co^2+^, which rendered electron density increase in the Ni center. NiCo-MOF achieved the best adsorption effect and catalytic capacity for LiPSs conversion as a result of synergistic effect. To be specific, the Ni-MOF showed the better catalytic effect on LiPSs conversion than Co-MOF, while Co-MOF exhibited a stronger interaction with LiPSs than Ni-MOF [105]. To realize effective adsorption and catalysis, Mai adjusted the metal sites by constructing bimetallic Zn/Co-ZIF (Fig. 7g). Specifically, Co site delivered better catalytic activity and Zn site could anchor LiPSs more strongly. By adjusting the Zn:Co ratio and using the Zn/Co-ZIF with a mole ratio of 0.9 as a separator modification layer, optimized cycling and rate performance of LSBs could be achieved. Its initial capacity was 1304 mAh g^−1^ and could maintain at 1141 mAh g^−1^ after 100 cycles [12]. The interaction between bimetallic MOFs and sulfur species can be optimized by adjusting the types of incorporated metal ions and tuning the electronic structure of metal sites with changed coordination environment. For example, a series of bimetallic MnM-MIL-100 (M = Co^2+^, Ni^2+^, Zn^2+^, Pb^2+^ and etc.) were synthesized, in which Co could modify the electronic structure of Mn, and the decrease in Ni content changed the electronic environment of Ni^2+^ [106].

##### Medium/High-Entropy TMCs

TMCs with more than two kinds of metal cations are also designed for LSBs. For example, Cu_2_ZnSnS_4_ with Cu, Zn and Sn as active sites was prepared to adsorb LiPSs and promote the redox reaction [107]. Considering the combination of various advantages of different TMCs and the cooperating effect of multi-metals, high-entropy materials (high-entropy alloys and high-entropy ceramics) containing more than five kinds of metal elements are also applied in LSBs. High-entropy materials with a single-phase crystal, possess significant advantages of lattice distortion and ‘cocktail’ effect, presenting a distinguished synergistic effect and incomparable catalytic activity [108]. Considering that Co-doped ZnS, CoS, and Cu-based materials are known catalytic materials for LSBs, Abruna combined Zn, Co, and Cu with In, Ga into the sulfide material to balance the charge, and designed high-entropy sulfide Zn_0.30_Co_0.31_Cu_0.19_In_0.13_Ga_0.06_S to facilitate the redox kinetic and alleviate the shuttle effect of LSBs. As a result, the high-entropy sulfide exhibited superior catalytic properties than any constituent metal sulfides in both discharge and charge stages. It was noteworthy that although GuGaS_2_ and CuInS_2_ had catalyzed the reduction reaction, no metal sulfides catalyzed the oxidation reaction, and the high-entropy sulfide showed significantly improved oxidation kinetics due to the synergistic interaction between the components of the high-entropy sulfide [109]. Qiao designed high-entropy oxide composed of highly dispersive Ni, Mg, Cu, Zn and Co, which exposed abundant active sites, and could strong anchor LiPSs with Li–O and S–Ni bonds and catalyze LiPSs conversion of (Fig. 6i) [110]. High-entropy metal nitride of V, Cr, Nb, Mo and Zr was also applied as sulfur host. The electron transferred from S_*x*_^2−^ (4 ≤ *x* ≤ 8) to high-entropy metal nitride, and the XPS peaks of X^3+^-N bond (X = Nb, Mo, Cr, V, and Zr) all shifted to lower binding energies with similar shift value, meaning the homogeneity and equality of those metals [111]. The (Mg_0.2_Mn_0.2_Co_0.2_Ni_0.2_Zn_0.2_)Fe_2_O_4_ nanofiber could provide large number of active sites for adsorbing LiPSs and synergistically promoting LiPSs conversion, Li_2_S deposition and decomposition, which was superior to that of NiFe_2_O_4_ and (Ni_1/3_Co_1/3_Mn_1/3_)Fe_2_O_4_ [112].

In addition, more than two kinds of metal ions can also be introduced into MOF materials to further enrich their active sites, improving their electrochemical properties. Pang designed FeCoNi prussian blue analogs (PBA), in which Fe, Co, and Ni could all act as binding sites for Li_2_S_4_ (Fig. 7h), achieving highest adsorption energy than that of FeCo-PBA and FeNi-PBA [113]. Furthermore, Pang constructed PBA ranging from binary to high entropy to explore the effects of different metal on the coordination environment, the mechanism of LiPSs conversion and the cathode performance. As a result, Fe coordinated with C, while Co, Ni, Cu, Mn and Zn all participated in N coordination. More importantly, high-entropy PBA delivered faster Li^+^ diffusion, minimum charge transfer resistance and faster LiPSs conversion, promoting the corresponding LSB to achieve the best cycle performance with an initial capacity of 1335.6 mAh g^−1^ and residual capacity of 570.9 mAh g^−1^ after 200 cycles at 0.1C, which was better than the LSBs with NiFe-PBA (1129.0 mAh g ^−1^, 339.5 mAh g ^−1^), NiCuFe (1169.1 mAh g ^−1^, 389.8 mAh g ^−1^), and CoNiCuFe (1218.3 mAh g ^−1^, 467.0 mAh g ^−1^) [114].

All in all, the study of high-entropy materials for LSBs is still in its infancy. Although high-entropy materials show better catalytic performance than their constituent components, the functions of each component, the synergies between each component and the mechanism of how to play the catalytic role are worthy of more in-depth discussion, which is conducive to the selection and matching of components of high-entropy materials to design efficiently multi-functional sulfur catalysts. For example, high-entropy alloy FeCoNiMnZn as catalyst had been shown to simultaneously improve the reaction kinetic for both in discharge and charge process of LSBs. This could be ascribed to the optimized electronic structure, that is, the *d*-band center of FeCoNiMnZn moved upward than that of FeCoNi and Zn, thereby increasing the adsorption energy and electrons transfer toward LiPSs. The electron density differences combined with partial projected density of states (PDOS) further displayed that Zn acted as an electron reservoir, Mn dominated the conduction band and promoted electron consumption, and other elements played the role in regulating charge distribution [115]. In addition, the chemical and structural stability of high-entropy materials in the charging and discharging process of LSBs should be considered. It was found that the copper in Zn_0.30_Co_0.31_Cu_0.19_In_0.13_Ga_0.06_S was leached out as an ionic species, accompanied with the smaller particles and lower crystallinity of high-entropy sulfide, gives a good indication that stabilizing cations could prolong the life of catalysts and improve the capacity retention ability [109]. Furthermore, the significant lattice distortion is also one of the key factors for the excellent catalytic performance of high-entropy materials. Nevertheless, how to control the lattice distortion of high-entropy materials and the relationship between it and catalytic activity has not been studied. Additionally, more high-entropy TMCs should be designed, such as high-entropy fluorides, high-entropy chlorides [116, 117].

Moreover, given that the composition range of high-entropy alloys is very large (1050 possible alloy components based on elements commonly used in the periodic table) and the traditional trial-and-error approach is too cumbersome for the design of high-entropy materials, high-throughput theoretical calculations combined with machine learning is an effective approach and has been widely used in the composition design and optimization of high-entropy catalyst in recent years [118, 119]. For example, using DFT calculations, Singh considered more than 1280 adsorption sites on the designed FeCoNiCuMo catalyst, developed three machine learning models to predict the adsorption of some important intermediates in the CO_2_ reduction process, and further analyzed the structure-performance relationship of catalysts to CO_2_ reduction [120]. Guo’s group proposed a first principles computational theory method for machine learning-aided design to study the oxygen reduction reactivity of millions of reaction sites on the surface of six kinds of high-entropy alloys, accurately predicting the catalytic activity of millions of reaction sites [121]. Luo and Chen revealed the atomic distribution, surface atomic structure and local coordination environment of high-entropy alloys based on machine learning, and further revealed the origin of highly efficient alkaline hydrogen oxidation reaction properties of high-entropy alloys combined with the DFT calculation [122]. Unfortunately, the research of high-throughput technology combined with machine learning has been rarely reported in the field of LSBs, which may be the future research hotspot of high-entropy materials applied to LSBs, due to the adsorption and catalytic mechanisms that can be used for reference.

#### Bi-anionic TMCs

**Fig. 8:**
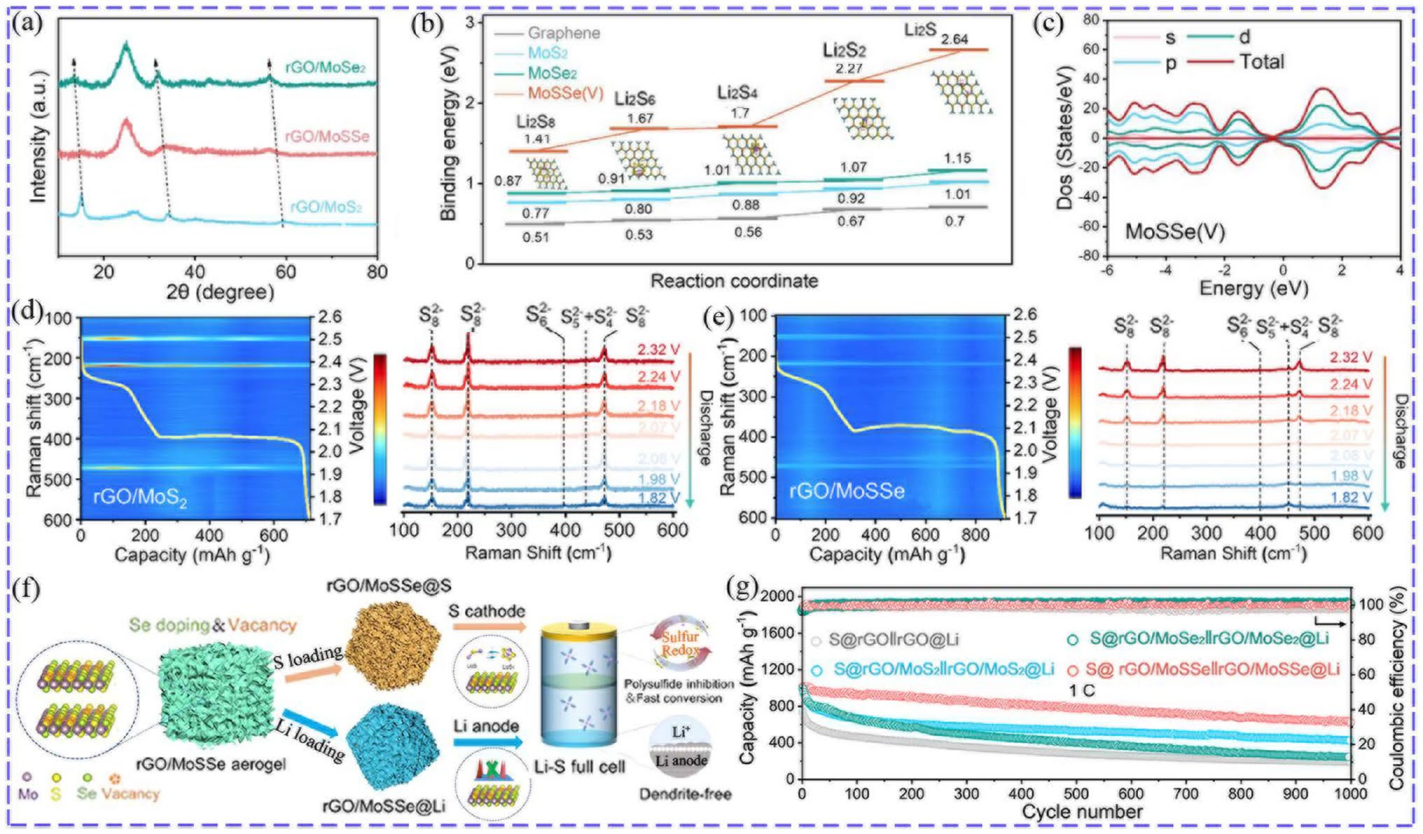
**a** XRD of rGO/MoS_2_, rGO/MoSSe and rGO/MoSe_2_. **b** Comparison of binding energies of LiPSs and MoS_2_, MoSe_2_, MoSSe (V). **c** DOS states. **d, e** In situ Raman characterization of cells with rGO/MoS_2_ and rGO/MoSSe. **f** Schematic of rGO/MoSSe as a host for both sulfur and lithium. **g** Cycling performance at 1C [124]. Copyright: 2022, American Chemical Society

Just like the implant of metal ions, TMCs with suitable dual-anions will also improve the conductivity and electrochemical activity [123]. Most importantly, the adsorption for LiPSs and the catalytic effect can also be well regulated by modifying the anions [29]. Zhou introduced Se into MoS_2_ lattice by anion substitution, which led to many anion vacancies and an increase in interlayer spacing due to the difference of radius and electronegativity of Se and S atoms (Fig. 8a). MoSSe delivered much higher binding energies (Fig. 8b) with sulfur species than MoS_2_ and MoSe_2_ and possessed higher conductivity with lowest bandgap (Fig. 8c). Furthermore, experiment results also demonstrated accelerated redox kinetics and alleviated LiPSs shuttle, as well as great lithiophilicity for lithium electrodeposition (Fig. 8d, e). As a result, MoSSe could simultaneously accommodate sulfur and lithium (Fig. 8f), and the corresponding full cell could achieve a discharge capacity of 637.3 mAh g^−1^ at 1C for 1000 cycles and stable cycle (Fig. 8g) [124]. The high binding energy of Li_2_S_2_/Li_2_S on MoN_*x*_ (7.34/4.66 eV) only allowed their easy deposition, while hindered the reversible catalytic conversion of LiPSs. Oxygen-modulated metal nitride (MoN_*x*_-O) were proposed to optimize the binding ability (4.54/4.33 eV for Li_2_S_2_/Li_2_S), effectively immobilizing and reversibly catalyzing LiPSs with the lowest decomposition energy barrier of 0.55 eV compared to MoO_*x*_ (0.66 eV) and MoN_*x*_ (0.91 eV) [125]. To conduct Li^+^ with S atoms and adsorb LiPSs with O atoms, Ce_2_O_2_S with O-Ce-S was considered to be more suitable than CeO_2_ with single O-Ce. Due to the additionally formed Li–S and S–S bonds between LiPSs and Ce_2_O_2_S, Ce_2_O_2_S presented a better adsorption effect for LiPSs and promoted LiPSs conversion with a higher conversion free energy compared to CeO_2_ [126].

BiOX (X = Cl, Br and I) with Bi located at outer surface of the molecular and X with larger electronegativity at inter, provided vacant orbitals of Bi as active sites and accepted electrons from LiPSs, showing excellent ability to adsorb LiPSs and catalyze their conversion [127]. Among them, BiOI was more suitable for its good conductivity with a smaller bandgap (1.9 eV) compared to BiOBr (2.9 eV) and BiOCl (3.5 eV). Furthermore, BiOI achieved better ability to capture LiPSs and bidirectionally promote their conversion. This could be proved by higher binding energies, smallest Tafel slope, larger nucleation capacity of Li_2_S, highest current and smallest polarization displayed in Li_2_S_6_ symmetrical cells compared with the others [128].

Because too strong interaction between Ti_3_C_2_ and S_8_/LiPSs caused the decomposition of sulfur species, Ti_3_C_2_ must be modified with surface terminating groups. Therefore, functionalized Ti_3_C_2_T_2_ (T = N/O/S/F/Cl) with *d*-*p* hybridization between Ti and T were studied as sulfur host. The binding energies were in the sequence of Cl < F < N < O < S, and the kinetic evaluation and Li^+^ diffusion got worse in the order of Ti_3_C_2_S_2_ > Ti_3_C_2_O_2_ > Ti_3_C_2_F_2_ > Ti_3_C_2_N_2_ > Ti_3_C_2_Cl_2_, which were closely associated with their interactions with sulfur species [129]. Compared with Ti_2_NO_2_ (2.07 eV taking Li_2_S as an example), Ti_2_NS_2_ (3.42 eV) possessed larger adsorption energies with sulfur species, which mainly provided by the interaction between Li atoms of LiPSs and S atoms of Ti_2_NS_2_. In addition, the Ti_2_NS_2_ exhibited metallicity that derived from the *d* orbital of Ti and the *p* orbital of S, and could maintain a high conductivity even after adsorbing LiPSs to facilitate the electrochemical reactions [18].

Hydroxyl oxides, a kind of compounds made up of oxygen ions, hydroxide ions and a metal ion, has also been applied in LSBs. Hydroxy iron oxides (FeOOH) with Fe–O, Fe–OH bonds and good conductivity were used to accommodate sulfur, in which transition metal and hydroxyl acted as adsorption sites for LiPSs and accelerated electron transfer between the catalyst and LiPSs, exhibiting good effect for capturing and catalyzing LiPSs [130, 131]. Similarly, MnOOH with Lewis acid sites Mn^3+^ cation could anchor LiPSs by forming Mn-S bond [132]. Moreover, the TMCs with dual-cations and dual-anions were designed. For example, LiVPO_4_F was applied to boost the kinetic of sulfur species, which provided both *p* orbitals and *d* orbitals of O, V, F, P atoms to interact with LiPSs [133].

In conclusion, compared to TMCs with single cation/anion, dual-ionic TMCs possess higher conductivity, better chemisorption effect and multiple active sites for catalyzing LiPSs conversion. However, the dual-ionic TMCs still lack in design basis, especially for the bidirectional catalysts. Additionally, there is a lack of research on the optimization of catalytic sites and catalytic activity by regulating the band structure, valence state, electron density of dual-ionic TMCs by adjusting the ions type, content, electronegativity, and ion radius difference. It is worth noting that the *d*-band center, spin polarization, and other electronic structures of dual-ionic TMCs can also be regulated by changing ion species, which is worthy of further exploration. Furthermore, although doped ions may not be able to form dual-ionic TMCs, and doping and dual-ionic TMCs are similar ways of modifying TMCs by introducing heterogeneous ions, the modification effects and mechanisms of the two should be compared, possibly by regulating the content of introduced heterogeneous ions. Furthermore, high-entropy materials with multi-anions are also of great interest but have not been widely studied [116]. Additionally, more modification methods should also be introduced to further improve dual-ionic TMCs, such as bimetallic TMCs quantum dots [134]. Bimetallic TMCs can also be combined with doping modification strategy to construct multi-functional sulfur hosts, such as N-doped CuCo_2_O_4_ [135].

### TMCs-Based Heterostructure Composites

**Fig. 9:**
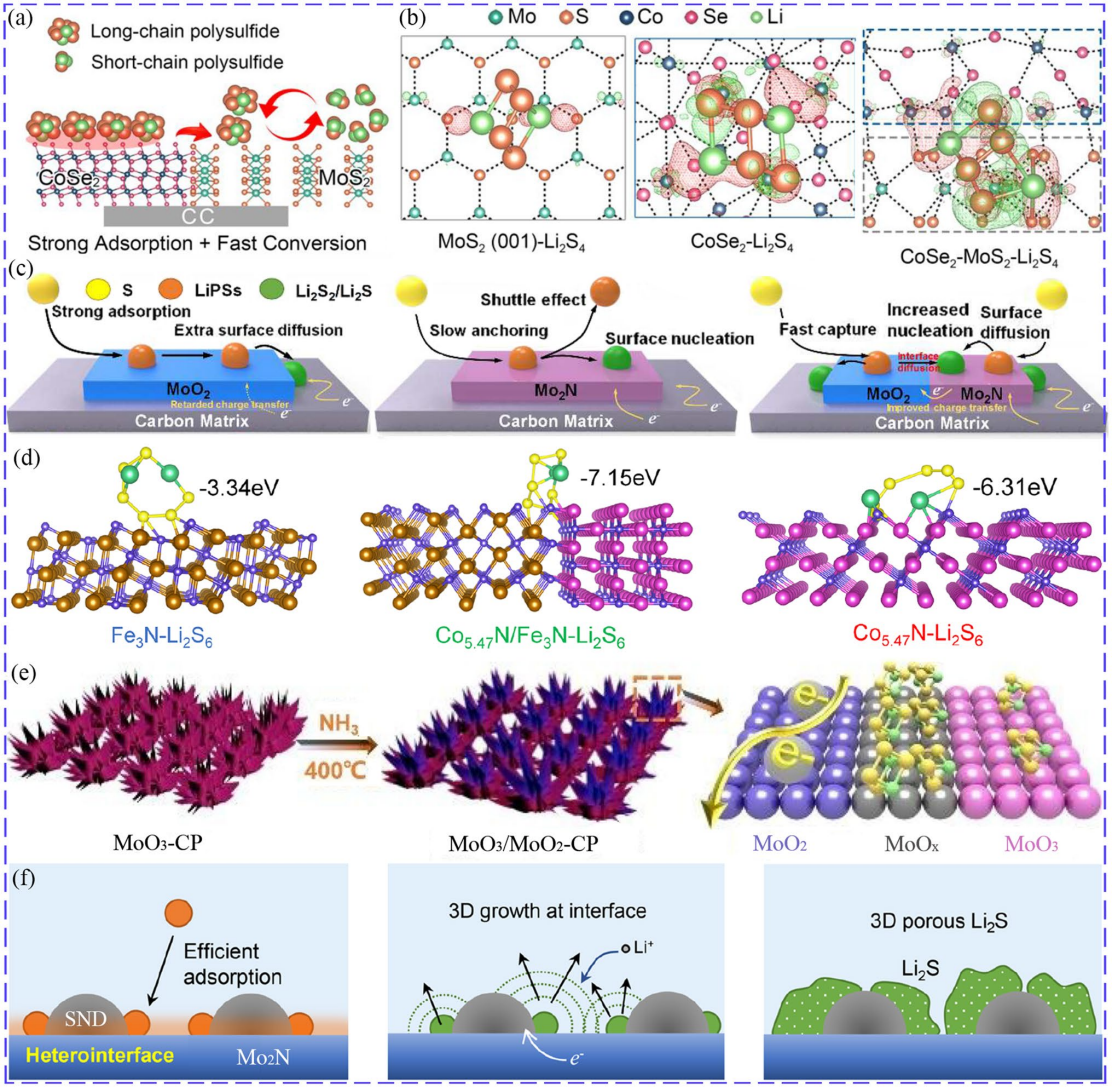
**a** Synergistic adsorption and catalysis of CoSe_2_/MoS_2_ heterostructure for LiPSs. **b** electron density differences of Li_2_S_4_ on MoS_2_, CoSe_2_ and CoSe_2_/MoS_2_ (the red and green regions represent negative and positive change [137]. Copyright: 2021, John Wiley and Sons. **c** Comparison of LiPSs conversion and Li_2_S precipitation on MoO_2_, Mo_2_N and MoO_2_–Mo_2_N [140]. Copyright: 2020, Elsevier. **d** Binding energies between Fe_3_N, Co_5.47_N/Fe_3_N, Co_5.47_N and Li_2_S_6_ [146]. Copyright: 2022, Elsevier. **e** Schematic diagram of MoO_3_/MoO_2_ [147]. Copyright: 2020, Royal Society of Chemistry. **f** Li_2_S growth at the SnO_2_–Mo_2_N interfaces in 3D model [149]. Copyright: 2021, American Chemical Society

When a kind of TMCs cannot simultaneously meet the needs of chemical binding with LiPSs and catalyzing their transformation, the heterostructure composites of two kinds of TMCs can be constructed with complementary advantages. For example, MoSe_2_ has excellent electrical conductivity and catalytic performance, but lacks sufficient chemisorption for LiPSs, while MoO_2_ has superior chemical binding effect. Therefore, the synergistic effect of efficient capture and catalytic transformation for LiPSs could be achieved with MoSe_2_/MoO_2_ heterostructure [136]. The edge sites of MoS_2_ possess high catalytic activity, while the basal planes have little activity. Zhang et al. grew CoSe_2_ on the base surface of MoS_2_ to form heterostructure, synergistically utilizing the superior chemisorption capacity of CoSe_2_ and the excellent catalytic activity of MoS_2_ edge sites (Fig. 9a). The CoSe_2_/MoS_2_ interface also had higher binding energy with LiPSs than CoSe_2_ and MoS_2_, which was confirmed by much more accumulated electrons at adsorption regions (Fig. 9b). As a result, compared to CoSe_2_ and MoS_2_, the LSB equipped with CoSe_2_/MoS_2_ delivered a most stable cycle performance with initial capacity of 825.5 mAh g^−1^ at 2C and retained capacity of 714.5 mAh g^−1^ after 600 cycles [137]. Similarly, Zhao et al. constructed Ni/Ni_2_P heterostructure to combine the strong adsorption of Ni_2_P for LiPSs and catalytic activity of Ni [138]. A kind of heterostructure composed of 0D bimetallic CoZn-Se nanoparticles on 2D nitrogen-doped MXene was designed, providing double lithiophilic–sulfiphilic binding sites through forming Co–S, Li–Se, Ti–S and Li-N/Li–O bonds to adsorb LiPSs and catalyze their conversion [139]. Typically, when one component had good adsorption performance and the other component possessed excellent catalytic activity, the adsorption–diffusion–catalysis process of LiPSs could be completed smoothly across the heterostructure interface, thus boosting the redox kinetic, as shown in Fig. 9c, such as MoO_2_–Mo_2_N [140], MoS_2_–MoN [141], and Nb_4_N_5_–Nb_2_O_5_ [142].

In addition to combining the advantages or complementing disadvantages of different TMCs, heterostructure composites can also balance the contradictions between them. Considering that too weak interaction is not conducive to interfacial charge transfer, and too strong interaction may hinder it, the chemical interaction should be moderate and balanced with the catalytic capacity. MoO_2_:Co_2_Mo_3_O_8_ heterostructure was prepared by doping Co, in which MoO_2_ possessed strong adsorption ability while Co_2_Mo_3_O_8_ delivered superior catalytic property and fast Li^+^ diffusion. To balance the interaction strength and catalytic capacity, the component of heterostructure composite was regulated by changing the cobalt content, and 9MoO_2_:2Co_2_Mo_3_O_8_ achieved the dynamic balance of adsorption-diffusion-conversion, delivering the best catalytic capacity for LiPSs conversion and the smallest Li_2_S nuclear barrier [143]. Similarly, the capturing capacity of WO_3_ and catalytic ability of WS_2_ were balanced in WS_2_–WO_3_ heterostructure by tuning the sulfurization degree of WO_3_, and the LSBs with 3WO_3_:1WS_2_ achieved the highest conversion efficiency [144].

Heterostructures composites can not only combine the advantages of two kinds of materials, but also possess the virtue of the interfacial effect, such as promoting charge transfer, diffusion of Li^+^ and LiPSs as well as adsorption capacity [145]. More importantly, the electronic properties of heterostructure can be optimized compared with the monomers, promoting the catalytic activity. The DOS of Co_5.47_N/Fe_3_N heterostructures clearly showed a higher DOS value at the Fermi level than that of Co_5.47_N and Fe_3_N, indicating a superior electrical conductivity. Additionally, Co_5.47_N/Fe_3_N presented the highest binding energy with Li_2_S_6_ (Fig. 9d), implying the synergistic effect at heterointerfaces [146]. Heterostructure interfaces also allow rapid diffusion of LiPSs from one component to the other. V_2_O_3_/V_8_C_7_ heterostructure was constructed to rapidly transfer the LiPSs strongly trapped by V_2_O_3_ to V_8_C_7_ through the interface, achieving efficient conversion [21]. MoO_3_/MoO_2_ heterostructure was synthesized by partially reducing MoO_3_ and transformed the non-conductive MoO_3_ into conductive MoO_2_, obtaining the defective MoO_*x*_ (2 ≤ *x* ≤ 3) interface with abundant oxygen vacancies (Fig. 9e), which presented greater adsorption capacity with binding energy of 1.78 eV for Li_2_S_4_ than that of MoO_3_ (1.13 eV) and MoO_2_ (1.00 eV). Benefitting from the different advantages of MoO_2_ and MoO_*x*_, the Li_2_S_8_ electrode with MoO_3_/MoO_2_ heterostructure achieved the best electrochemical properties, which could retain a capacity of 828.1 mAh g^−1^ at 0.5C after 500 cycles with a capacity decay rate of 0.016%, in contrast to the MoO_3_ with 0.070% and MoO_2_ with 0.083% [147]. In addition, heterostructure interface can also provide Li_2_S nucleation sites, facilitate the nucleation and regulate the three-dimensional growth of Li_2_S to avoid the passivation of catalyst surface, increasing deposition capacity [148]. Lv revealed that Li_2_S precipitated nonuniformly on the surface of Mo_2_N with thick layer and a rough morphology. Surprisingly, when small SnO_2_ nanodots anchored on Mo_2_N microbelt and formed highly active heterointerfaces, the growth of Li_2_S was guided in a 3D model, avoiding surface passivation of the catalyst (Fig. 9f) [149].

**Fig. 10:**
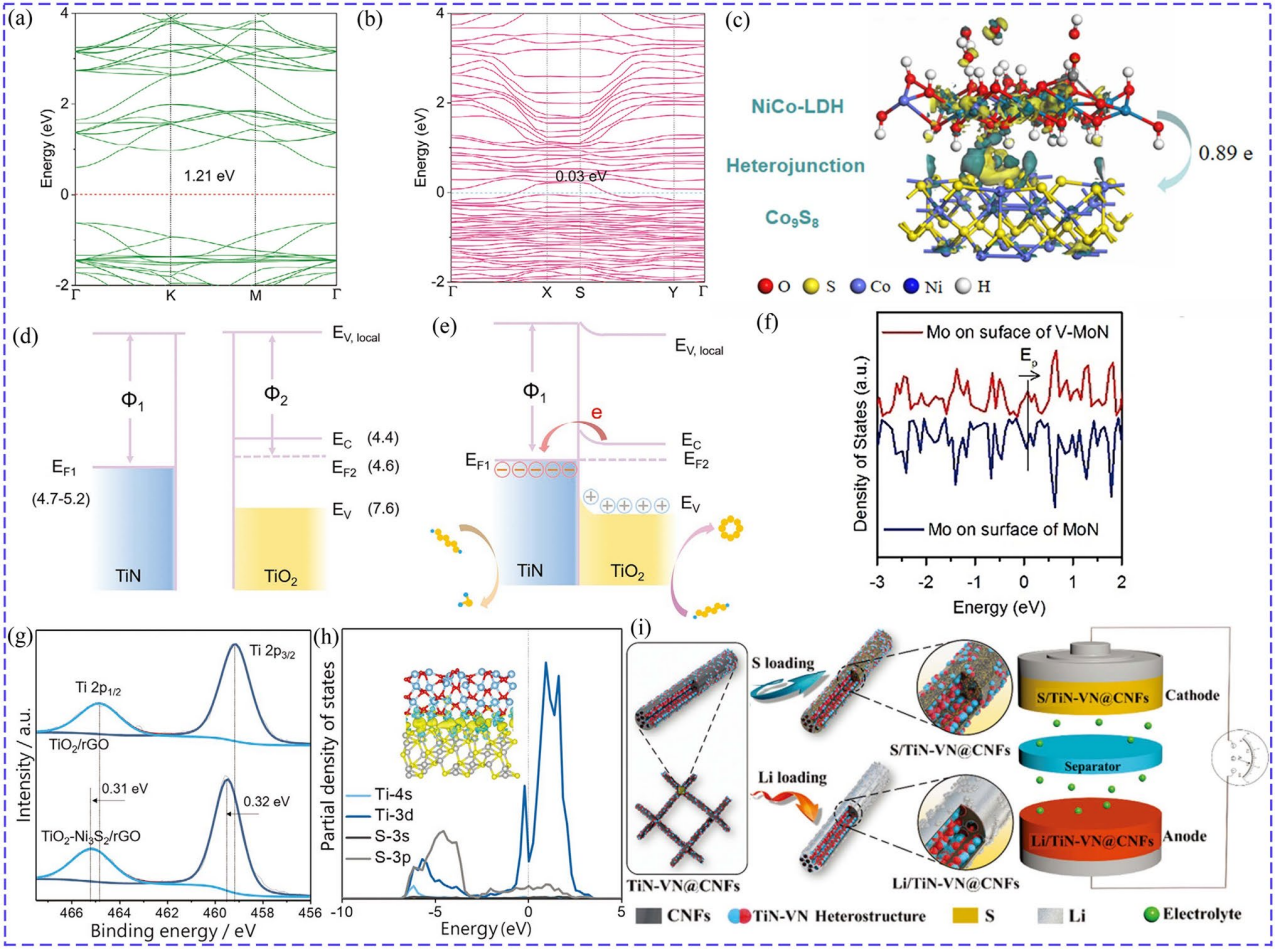
Band structure of **a** ZnSe and **b** CoSe–ZnSe [151]. Copyright: 2021, John Wiley and Sons. **c** Electron density difference of NiCo-LDH/Co_9_S_8_ [153]. Copyright: 2020, Elsevier. Energy band of TiN and TiO_2_ **d** before and **e** after placing in contact [158]. Copyright: 2020, John Wiley and Sons. **f** DOS of Mo of V-MoN and MoN [159]. Copyright: 2018, John Wiley and Sons. **g** XPS of Ti 2*p* peaks of TiO_2_/rGO and TiO_2_-Ni_3_S_2_/rGO. **h** PDOS of Ti and S atom of the Ni_3_S_2_/TiO_2_ interface [164]. Copyright: 2020, John Wiley and Sons. **i** Schematic diagram of TiN-VN@CNFs used as both Li host and S host to construct full battery [165]. Copyright: 2019, John Wiley and Sons

Heterostructures composites with enhanced adsorption and catalytic ability can improve cathode performance compared to their components, due to higher electric conductivity with optimized energy band structures (Fig. 10a, b), boosted interfacial charge transfer kinetics, accelerated ion diffusion, and more active sites [150, 151]. More importantly, the heterostructures induce internal electric fields, and the electron is redistributed as electron transfer occurs and local atoms are arranged [152, 153], such as NiCo-LDH/Co_9_S_8_ (Fig. 10c). ZnS–FeS heterostructure was designed with large energy bandgap offset between ZnS (intrinsic energy bandgap of 4.9 eV) and FeS (0.8 eV). The strong built-in electric field at the heterointerface could effectively promote the charge transfer in redox reaction, thus improving catalyzing capability for LiPSs conversion [154]. Electrons redistribution occurs at the binary interface and the electronic structure of the TMCs is regulated, elevating the catalytic activity and chemisorption ability [53, 155].

It has been proved that it is the Fermi energy levels difference of two materials that caused built-in electric field and electrons transfer through the interface [156]. A Mott–Schottky heterostructure was constructed with positively charged CoFeP and negatively charged C_3_N_4_. The difference in Fermi levels rendered electrons transfer from C_3_N_4_ to CoFeP to balance their work function at the interface. As a result, the energy bands of C_3_N_4_ upward bended at the interface and an electric field formed from C_3_N_4_ to CoFeP. The charge was redistributed at the interface, promoting the catalytic activity and Li^+^ diffusion [157]. A Mott–Schottky catalysts (TiON) was also built by spontaneous oxidation of TiN due to the lower work function of TiO_2_ compared to TiN (Fig. 10d). The charge redistribution at the interface could promote the adsorption and catalytic ability (Fig. 10e) [158]. Qiao designed MoN-VN heterostructure, which achieved higher adsorption capacity for LiPSs than MoN, due to the regulated electronic structure. To be specific, the E_p_ position (nearest peak to Fermi level) of the Mo of MoN-VN became higher (Fig. 10f), resulting in higher antibonding states compared with MoN [159]. This was because the higher E_p_ position, the lower antibonding orbital occupancy, leading to stronger interaction between LiPSs and catalysts [160]. The theoretical calculations for Co_3_S_4_/MnS also showed that the introduction of Mn tuned the electronic structure of Co_3_S_4_ with higher E_p_ position, contributing to more effectively adsorb LiPSs, facilitate charge transport and improve the conversion kinetics [161].

Considering that different TMCs can catalyze the reaction in different stages of LSBs, heterostructure composites can comprehensively facilitate the reaction kinetics with different catalytic activities of various TMCs. CoO/NiO heterostructure was constructed, in which NiO could accelerate the solid–liquid conversion, and CoO was more conducive to improving the liquid–solid reaction kinetics [162]. For MoSe_2_@F-doped carbon@Mo_2_C heterostructure, MoSe_2_ and Mo_2_C selectively catalyzed the conversion of long-chain LiPSs, and F-doped carbon tended to catalyze the reduction of short-chain LiPSs, realizing multi-step catalysis [163]. Heterostructure composites can also be used as bidirectional catalyst. It was proved that a CoSe–ZnSe heterostructure could not only accelerate sulfur reduction with a reduced energy barrier of 0.43 eV compared to ZnSe (0.54 eV), but also facilitate Li_2_S decomposition with a lower oxidation energy barrier of 0.93 eV than that of ZnSe (1.04 eV), thus bidirectionally catalyzing sulfur conversion reactions [151]. Yang et al. designed a TiO_2_-Ni_3_S_2_ heterostructure, in which strong electronic interactions was occurred between TiO_2_ and Ni_3_S_2_ (Fig. 10g), due to the hybridization between the *p* orbital of S atom of Ni_3_S_2_ and the *d* orbital of Ti of TiO_2_ (Fig. 10h). TiO_2_-Ni_3_S_2_ possessed bidirectional catalytic effect, that is, TiO_2_ captured LiPSs and Ni_3_S_2_ catalyzed LiPSs conversion during the reduction process, and both of them showed catalytic activity for Li_2_S oxidation during charging process [164]. Moreover, TiO_2_-Ni_3_S_2_ as well as TiN-VN could serve as not only sulfur hosts to boost the redox of sulfur cathodes, but also lithium host to facilitate uniform lithium deposition and significantly inhibit dendrites, thus matching Li–S full-batteries with superior electrochemical performance (Fig. 10i) [165, 166].

Until now, plentiful heterostructure composites have been explored, especially constructed by TMCs with same anion but different metal ions (such as MoS_2_/Ni_3_S_2_ [167]) or same metal ion but different anions (such as Mn_3_O_4_-MnP_*x*_ [168], Fe_9_S_10_/Fe_3_O_4_ [169]). For TMCs-based heterostructure materials composed of different anion and cation, one of the compounds can be prepared first, and then another metal compound can be grown on its surface. Taking SnO_2_–Mo_2_N as an example, Mo_2_N was firstly synthesized and dispersed in water, which was then mixed with SnCl_2_·2H_2_O in a mixed solution of HCl and water, and SnO_2_–Mo_2_N was obtained by heat treating the mixture in Ar atmosphere. Heterogeneous composites with the same anion and different metal ions can be prepared by adding different metal sources at the same time, and then hydrothermal or solid phase sintering treated according to the state of the raw material (aqueous solution or solid state), and the product type can be controlled by controlling the proportion of raw materials. The MoO_2_: Co_2_Mo_3_O_8_ heterostructure was obtained by drying the mixture of the ammonium molybdate solution and cobalt acetate solution, and then sintering in N_2_ [143]. For heterostructures with different anions of the same metal, single-component metal compounds can be prepared first, and then prepared by adjusting the sintering atmosphere, temperature and time, or sintered with chemical reagents containing the anions at high temperatures, such as adding thiourea to prepare sulfide heterojunctions. For example, MoO_2_-Mo_2_N was prepared by annealing the prepared MoO_3_ in NH_3_ atmosphere and passivating in O_2_/N_2_ [140]. Similarly, precursors containing anions of heterostructure material are synthesized, and then to prepare the heterostructure with different anions using the resulting gas during high-temperature sintering. For example, MoO_2_, MoO_2_/MoC and MoC were synthesized by annealing the synthesized precursor Mo_3_O_10_(C_6_H_8_N)_2_·2H_2_O in Ar for 4 h at different temperatures of 600, 650, and 700 ℃, respectively [149].

Specifically, a kind of organic–inorganic heterostructure consisting of covalent triazine framework and Ti_3_C_2_ MXene was constructed with covalent interfacial interaction due to the formed Ti-N bonds. This composite was imparted with dual adsorption sites supplied by lithiophilic N and sulfurophilic Ti [170]. In conclusion, despite the respective advantages of heterostructure components are clearly studied, the catalytic effect and its mechanism of heterostructure interface itself should be further deeply studied. Long grew Pt particles on NbC surface to bifunctionally catalyze the redox of sulfur cathodes. Specifically, NbC was likely to adsorb LiPSs and Pt promoted their conversion, while both NbC and Pt could catalyze Li_2_S decomposition [171]. However, in their another study, the mixture of Pt and Nb_2_O_5_ without heterostructure interface could also play bifunctional catalytic roles similar to Pt and NbC [172]. Similarly, the mixture of metal-based catalysts without heterostructure could also improve the adsorption ability and catalytic effect, such as Co mixed with Mo_2_C [173], Co@CoO core–shell structure [174], TiO_2_@TiN [175]. Unfortunately, the roles of the heterostructure interfaces and their catalytic mechanism are unclear. Moreover, the effect of different components on the catalytic ability of heterostructures should be studied, such as MO_*x*_-MXene (M: Ti, V and Nb) heterostructures [176]. And the types of the interface are lack of regulation and study, such as coherent interface or semi-coherent interface [177], amorphous/crystalline heterostructure [178]. In addition, the content regulation and heterostructure distribution should be paid more attention, such as continuous interface [179]. And the heterostructures constructed with more than two materials has not been developed in LSBs, such as ternary heterostructures, which are of great significance for well-designed sulfur catalysts [180]. Meanwhile, the morphology of heterostructure materials should also be considered, such as lychee-like TiO_2_@TiN hollow spheres [181], Co-Co_3_O_4_ hierarchical array nanostructure [182].

## Conclusion and Prospects

In conclusion, the research progress of the superiorities of TMCs modified with multiple cations/anions and TMCs-based heterostructure composites as catalysts in LSBs have been comprehensively discussed. The main points are summarized as follows:

1. The three kinds of modification strategies all can boost the catalytic effect by providing more active sites and regulating the electronic structure.
2. Electron redistribution occurs between different components (doped ions or TMCs) due to the electron transfer with the introduction of additional ions or compounds to regulate the electronic structure, including energy band structures, *d*-band center, electron filling, and valence state for different electronegativity, *d* electron number and introduced vacancies, lattice distortion.
3. The superiorities of modified TMCs include better electronic/ion conductivity, enriched or optimized active sites, stronger chemisorption ability, enhanced catalytic activity, smooth LiPSs diffusion, regulated Li_2_S precipitation and so on. The balance and optimization of the adsorption strength and catalytic activity for LiPSs can also be achieved by optimizing the types of multiple ions.
4. Considering that different components play different functions of adsorption or catalysis for various sulfur species or reactions in each charging and discharging stages, selecting appropriate cations/anions of TMCs can construct multi-functionally bidirectional catalysts.
5. Synergistic effect exists in different components of doped TMCs, dual-ions TMCs, TMCs heterostructure composites. However, the synergistic mechanism is unclear.

These advantages eventually endow LSBs with improved cathode performance, as summarized in Table 1, including specific capacity, cycle stability and rate performance.

**Table 1:** A brief summary of the improvement of cathode performance by different modified TMCs catalysts

Catalysts	Current density (C)	Initial capacities (mAh g^−1^)	Cycling number	Decay rate (%)	Promoted mechanism	References
Ni-WS_2_ WS_2_	0.2	1160.8 963.5	100	54.5 55.1	More chemical anchoring sites, enhanced catalytic activity with surface defect	[36]
Ni-MoS_2_ MoS_2_	0.2	1343.6 1287.8	100	59.5 52.7	Better adsorption ability, increased catalytic activity more active sites	[39]
Ni_0.2_Mo_0.8_N Mo_2_N Ni_3_N	1	1280.8 1130 1072	1400 1400 800	36.4 31.7 29.1	Expanded lattice spacing, In situ etching polysulfide and generating vacancies	[40]
Fe(0.1)/Co_3_O_4_ Fe(0.2)/Co_3_O_4_ Co_3_O_4_	0.2	1392.6 – –	150	73.1 53.91 43.13	Multi-shelled structure, rich oxygen-defect	[54]
N/CoSe_2_ CoSe_2_	0.2	1341 1159	250	68.9 53.1	New defect, closer d-band center, higher charge number of Co, shorter Co–S bonds and weakened S–S and Li–S bonds	[59]
NiSe_2_ P-NiSe_2_	1	1012.5 931.7	500	72 61.5	Higher electron densities, enhanced electron transfers	[62]
P-NiTe_2-*x*_ NiTe_2-*x*_ NiTe_2_	0.2	1309 1270 1207	300	86.2 – –	Bonds reconstruction, electron densities redistribution	[61]
NiCo-LDH-Se-1 NiCo-LDH-Se-2 NiCo-LDH-Se-4	2	– 1332 –	1000	31.6 80.3 74.9	Improved conductivity, optimized electronic structure, abundant active site	[65]
V_2_O_5_ LiV_3_O_8_	0.1	1162 1254	100	69.5 77.3	Improved adsorption ability	[81]
TiO_2_ BaTiO_3_	0.2	913 941	120	79.5 91.2	Self-polarization of BaTiO_3_, strong interaction with LiPSs	[87]
Ni_3_ZnC_0.7_ Ni_3_C	1	1275.8 934.6	200	67.4 –	Extra lithiophilic sites of Zn	[90]
HEO CNF	1	879.6 544.2	500	63.5 –	Synergistic effect of multiple cations, abundant active site	[99]
NiCo-MOF Ni-MOF Co-MOF	0.1	974 761 638	80	92 – –	Different catalytic function of Ni and Co, charge redistribution	[12]
FeNi-PBA FeCo-PBA FeCoNi-PBA	0.1	1303.2 1029.4 1234.7	100	15.9 36 36.5	Multi-metal synergistic adsorption	[109]
CoSe_2_/MoS_2_ CoSe_2_ MoS_2_	0.1	1425.3 1092.4 1288.4	50	84.6 64.1 67.8	Stronger adsorption ability of CoSe_2_, higher catalytic activity of MoS_2_	[126]
Ni Ni/Ni_2_P Ni_2_P	1	721.3 836.1 770	500	60.2 76.1 72.1	Enhanced conductivity, charge transfer, and adsorption	[127]
MoS_2_ MoN MoS_2_–MoN	0.2	– – 1100	100	64.9 81.4 93.9	Catalyzing LiPSs by MoN, promoting Li^+^ diffusion by MoS_2_	[130]
CoSe–ZnSe ZnSe	0.2	1260 –	100	74.8 45.1	Charge redistribution and lattice distortion of heterointerface	[140]
V_8_C_7_–VO_2_ V_8_C_7_	4	765.3 666.9	900 500	45.1 43.5	Better anchoring ability, lithiophilic nature	[155]
TiO_2_/TiN TiO_2_	0.3	1397 1177	150	58 59.5	Adsorptive TiO_2_, catalytic TiN, charge transfer from TiN to TiO_2_	[169]

The *d*-band center of the metal sites is widely considered to be an effective descriptor of adsorption strength and catalytic activity as the metal sites are the catalytic center. The shift of the *d*-band center of metal toward the Fermi level increases the probability of electrons filled in the antibonding orbital between metal and adsorbed molecules, which boosts the adsorption ability for sulfur species, while the decrease in *d*-band center weakens the adsorption strength. Moreover, the relationship between catalytic performance and adsorption strength presents volcano plot. In other words, the adsorption strength is enhanced with the increase in *d*-band center in a certain range, and too strong adsorption is not conducive to the LiPSs conversion. On the other hand, smaller *d*-*p* gap means a reduced energy gap between the bonding and antibonding orbitals, thus facilitating the interfacial electron transfer and the conversion of LiPSs. However, the upward shift of *d*-band center does not mean that the *d*-*p* gap becomes larger, because the introduction of metal ions into TMCs also adjusts the *p*-band center [183]. Therefore, for TMCs containing anions, considering that the energy of the electrons in the *p* and *d* orbitals will directly affect bond formation and breaking, the *p*-band center of the nonmetallic site should be considered along with the *d*-band center.

At present, modification with metal ions has been extensively studied, including the factors that improve the catalytic performance (such as *d*-band center, *d*-band electron filling, valence state, vacancies, and lattice distortion) and the corresponding characteristics of doped ions, i.e., electronegativity, *d* electron numbers, electron donating ability, doping amount, doping site. Nevertheless, anion modification is not as widespread as cationic modification, and there is still a large space for anions modification. And the other electron structure such as spin polarization, *p*-band center *d*-*p* gap, are also of great significance need to be discussed. Most importantly, the standard descriptor for an excellent catalyst should be determined. Furthermore, the structure–activity relationships between electronic structure, crystal structure and catalytic effect of catalysts are worth further study. Taking *d*-band center as an example, considering that too strong adsorption will hinder the further transformation of LiPSs, the appropriate ranges of the *d*-band center of the catalyst with the best catalytic activity and the corresponding binding energies as well as surface diffusion properties for various LiPSs should be given, to better compare catalyst performance and facilitate the design of catalysts for LSBs. In addition, the influence of characteristics of the introduced ionic elements on the catalytic effect should be quantified. For example, the quantitative relationship between electronegativity values should be studied in depth, *d* electron numbers of metal elements, *p* electron numbers of nonmetallic elements and catalytic performance, rather than merely showing that they have an effect on the catalytic activity. Different modifications may present different advantages, so composite modifications should be developed to comprehensively improve the performance of sulfur catalysts. This can include constructing compounds with both multi-metals and multi-anions, doping heterostructure composites, compositing bimetallic TMCs and heterostructure, such as N-doped Co_2_VO_4_-Co heterostructure [184].

Challenges remain in designing sulfur cathode materials to meet all the requirements. Therefore, it is essential to make improvements in the following aspects. (i) Strengthen the study of the effect of regulating components on the electronic/crystal structure of TMCs, and the structure–activity relationship between the electron/crystal structure and the catalytic effect of sulfur cathodes. (ii) Deepen the understanding of the catalytic mechanism in different charge and discharge stages and the relationship between chemisorption and catalysis. (iii) Pay more attention to the analysis of roles for different components and the establishment of material selection criteria. (iv) To construct multi-components catalytic materials with synergistic effect, theoretical studies are considered a highly effective approach to understand how each catalytic component works, what electronic and/or chemical properties of catalyst components play the role, and the synergistic mechanism from the atomic level, reasonably guiding the selection and match of multi-components [36]. Furthermore, considering the abundance of elements and huge space to engineer the TMCs or high-entropy catalysts, machine learning should be developed to help design perfect catalysts meeting all the criteria.
